# Too Hot, Too Wet: Bayesian Spatial Modeling of Climate‐Driven Salmonella Risk in New South Wales, Australia, 1991–2022

**DOI:** 10.1029/2025GH001617

**Published:** 2026-01-28

**Authors:** Oyelola A. Adegboye, Tehan Amarasena, Mohammad Afzal Khan, Hassan Ajulo, Anton Pak, David Taniar, Theophilus I. Emeto

**Affiliations:** ^1^ Menzies School of Health Research Charles Darwin University Darwin NT Australia; ^2^ Public Health and Tropical Medicine College of Medicine and Dentistry James Cook University Townsville QLD Australia; ^3^ Faculty of Information Technology Monash University Melbourne VIC Australia; ^4^ Centre for the Business and Economics of Health The University of Queensland Brisbane QLD Australia

**Keywords:** Salmonella, foodborne disease, climate variability, environmental health, climate change and health, spatial bayesian

## Abstract

*Salmonella* infections contribute significantly to gastrointestinal‐related hospitalisations in Australia and remain a major global public health concern. Although seasonal patterns in *Salmonella* incidence have been documented globally, there is limited evidence on the influence of climatic factors, particularly rainfall, humidity, flooding, and temperature, in the Australian context. This study investigated the relationship between climatic extremes and *Salmonella* infections across Local Health Districts (LHDs) in New South Wales (NSW), Australia, using a Spatial Bayesian Distributed Lag Non‐Linear Model. Spatial modeling revealed a marked geographical heterogeneity in the risk of *Salmonella* related to climate in NSW. High ambient temperatures consistently increased risk, with 99th‐percentile contrasts typically yielding relative risks (RR) of 2.4–4.8 across LHDs. Monthly rainfall showed the opposite direction statewide: very dry months were associated with a higher risk, whereas very wet months were generally protective (RR<1). In contrast, discrete flooding events were strongly and positively associated with risk (99th‐percentile flood index RR ∼18–23.5), with the greatest effects in some LHDs of the metropolitan/coastal region. Humidity displayed modest but consistent positive associations (99th‐percentile RR ∼1.1–1.5). Temperature and humidity exhibited J‐shaped exposure‐response relationships, where the lowest risk occurred at moderate values. This contrasts with rainfall, which demonstrated an inverse (protective) association, and flooding, which showed a monotonic increase in risk with intensity. These results have important public‐health implications under a warming, flood‐prone climate.

## Plain Language Summary

*Salmonella* is a major cause of stomach illness and hospitalization. We examine how temperature, rainfall, humidity, and floods relate to *Salmonella* in New South Wales (NSW), Australia. Hot periods were associated with a higher risk, while high humidity contributed to a smaller increase. Regular heavy rainfall tended to be protective on average, but discrete flooding events were strongly associated with higher risk, likely due to contamination from sewer and stormwater overflow. These findings suggest that early warnings and targeted public‐health actions are most useful during heatwaves and floods.

## Key Points

- J‐shaped relationships were observed for temperature and humidity, with the lowest risk at mild conditions
- Spatial modeling revealed significant geographic variation, with inland and rural regions experiencing greater increases in risk
- Flooding and humidity were also associated with elevated *Salmonella* risk, especially in coastal and arid regions

## 1 Introduction

Salmonellosis, the infection caused by *Salmonella* bacteria, contributes significantly to the global burden of disease, with an age‐standardized mortality rate of 3 per 100,000 population (Ikuta et al., 2022). Transmission occurs by consuming contaminated food (e.g., undercooked meat, eggs, dairy, raw fruits, and vegetables), drinking contaminated water, coming into contact with infected persons' feces or infected animals (e.g., cattle, chickens, rodents, tropical fish and reptiles) (NSW Health, 2024; World Health Organization, 2018b). The incubation period can vary from 6 to 72 hr, and individuals are considered infectious while their stools test positive for *Salmonella* (NSW Health, 2024; World Health Organization, 2018b). A person can potentially be infectious for days to weeks after acquiring the infection (NSW Health, 2024). In developed countries such as Australia, it is usually less likely to be fatal, typically manifesting as an acute gastroenteritis (Darby & Sheorey, 2008). However, it can spread rapidly and still cause severe illness, particularly in vulnerable populations with underlying health conditions. In 2015, *Salmonella* was associated with an estimated 3,013 hospitalizations and 13 deaths, resulting in a societal cost of over $100 million (Akil et al., 2014).

*Salmonella*, a leading cause of food‐borne gastroenteritis and sepsis, has been associated with a rising incidence of foodborne illness in Australia (Ford et al., 2016; Ratnayake et al., 2024). National Notifiable Diseases Surveillance System data show that notifications almost doubled from 9,058 cases in 2009 to a peak of 18,044 in 2016, before falling sharply during the COVID‐19 period to 10,129 in 2022 and rebounding modestly to 11,205 in 2023 (Australian Government Department of Health and Aged Care, 2024). The pattern in New South Wales (NSW) mirrors the national trend: notifications climbed from 2,651 in 2009 to 4,519 in 2016 and have since declined by about one‐third, stabilizing at about 3,031 cases in 2023 (Australian Government Department of Health and Aged Care, 2024). Despite this recent decrease in notifications, current counts remain higher than a decade ago, underscoring the continuing burden.

A growing international literature demonstrates that *Salmonella* transmission is climate‐sensitive, exhibiting seasonal variation (Akil et al., 2014; Manchal et al., 2024; MJiang et al., 2021; Tack et al., 2021; Thindwa et al., 2019; Zhang et al., 2010). Time‐series studies in the Democratic Republic of Congo and Malawi have linked heavy rainfall and elevated ambient temperature, respectively, to surges in invasive non‐typhoidal *Salmonella* (Tack et al., 2021; Thindwa et al., 2019), while warmer, wetter months are associated with faster bacterial replication and increased case counts, particularly in the southern United States (Akil et al., 2014).

Australian evidence, though sparse, points in the same direction. In Adelaide, a 1°C increment in warm‐season temperature increased notifications by 1–4% (Milazzo et al., 2016a), and a national study covering 1991–2019 found that a 1°C rise in mean monthly temperature anomaly was associated with a 3.4% increase in cases, with El Niño periods elevating risk in NSW and Queensland (Davis et al., 2022). NSW is highly exposed to hydrometeorological hazards such as floods, bushfires, cyclones and hailstorms, which cost the state an estimated $5.1 billion in 2020–21 and are projected to escalate under climate‐change scenarios (NSW Treasury, 2021).

International and Australian studies consistently associate higher temperatures—and in some settings heavy precipitation—with increased salmonellosis (Akil et al., 2014; Davis et al., 2022; Manchal et al., 2024; Milazzo et al., 2016a; MJiang et al., 2021; Tack et al., 2021; Thindwa et al., 2019; Zhang et al., 2010). For Australia, city‐ and national‐level analyses report positive temperature effects and ENSO‐related variability (Davis et al., 2022; Milazzo et al., 2016a; Zhang et al., 2010). Spatial heterogeneity in NSW serotypes further motivates small‐area modeling (Simpson et al., 2019). However, most prior work either examines single exposures, uses linear/threshold forms, or lacks explicit spatial pooling—leaving open questions about non‐linear exposure–lag responses and geographic variation across NSW Local Health Districts (LHDs).

Distributed lag non‐linear models (DLNMs) flexibly capture non‐linear, delayed exposure–response relationships (Gasparrini et al., 2010). Recent advances extend DLNMs into a *spatial* Bayesian framework (SB‐DLNM), enabling partial pooling of exposure–lag curves across small areas while accounting for spatial correlation via intrinsic conditional autoregressive (iCAR) priors (Besag et al., 1991; Lawson, 2018; Quijal‐Zamorano et al., 2024). This approach improves the stability of local estimates and yields coherent maps of risk and exceedance probabilities. Building on these developments, we fit spatial Bayesian DLNMs to NSW LHD data to estimate LHD‐specific climate–*Salmonella* associations. Bayesian frameworks are also widely used in hydrology for streamflow prediction and uncertainty quantification, relevant here because our flooding indicator derives from high streamflow. For example, Bayesian model averaging (including copula‐embedded variants) and ensemble learning to combine signals across models (Sattari et al., 2024; Wang et al., 2025).

However, the combined influence of rainfall, temperature, humidity, and discrete flood events has not been examined at finer spatial scales, and no study has systematically assessed these drivers across NSW LHDs. Therefore, this study aims to evaluate the influence of key meteorological factors, including temperature, rainfall, and discrete flood events, on the incidence of *Salmonella* infections in NSW, Australia. Building on limited but consistent national evidence linking temperature with enteric disease, this study addresses a critical gap by integrating multiple climate exposures and examining their effects at the LHD level, using spatial and temporal data. The study seeks to identify geographic disparities in climate‐related enteric risk and to assess whether metropolitan and regional LHDs exhibit differential vulnerabilities. Ultimately, the findings aim to inform climate‐resilient food safety policies, strengthen early‐warning surveillance systems, and provide a scalable framework for enteric disease preparedness in the face of escalating hydro meteorological hazards under future climate change scenarios.

## 2 Materials and Methods

### 2.1 Study Area and Data Sources

New South Wales is Australia's most populous state, located on the southeast coast and spanning coastal plains, the Great Dividing Range, tablelands, western slopes, and extensive inland plains. The state is administratively organized into 15 LHDs, comprising six metropolitan LHDs centered on Greater Sydney and the coast, and nine rural/regional LHDs that cover agricultural districts, rangelands, and remote arid areas. Population density is highly uneven, with large urban centers (Sydney, Illawarra, Central Coast, Newcastle) concentrated along the coast and lower‐density settlements across the interior. This urban–rural gradient aligns with differences in infrastructure (e.g., reticulated water and sewage in metropolitan areas vs. mixed systems, including septic and rainwater, in some regional communities) and food supply chains, both of which can influence enteric disease risks.

**Figure 1:**
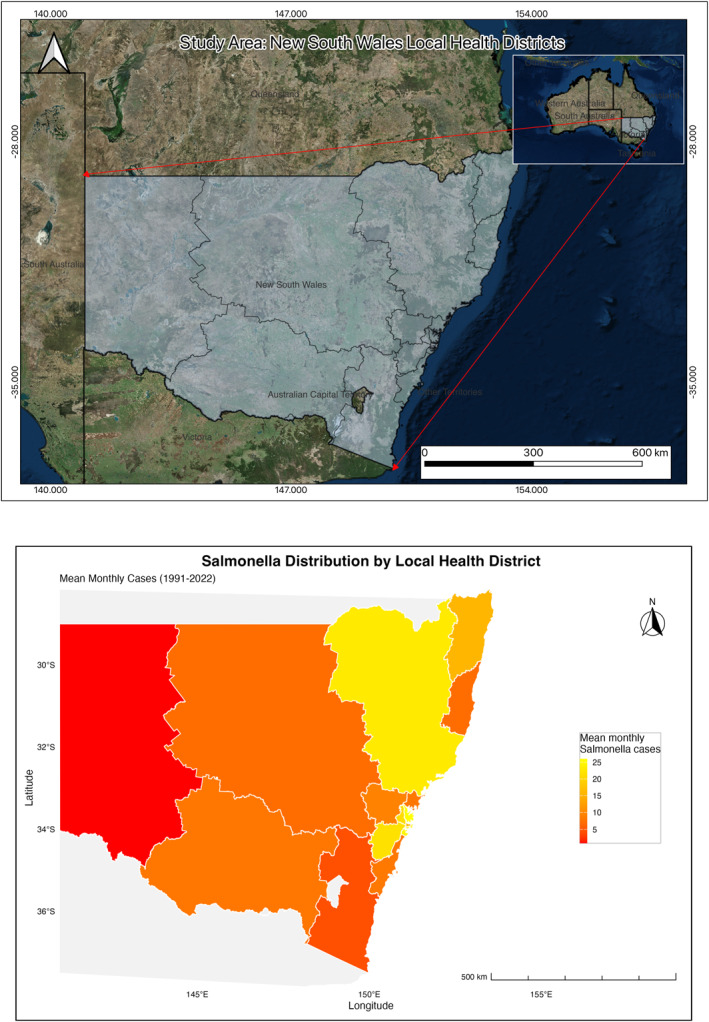
(Top) Map of Australia and the study area (bottom) Mean monthly *Salmonella* cases by Local Health District, 1991–2022 (counts; not population‐standardized).

Climatically, NSW encompasses several Köppen zones ranging from humid subtropical on much of the coast, temperate conditions on the tablelands, and semi‐arid to arid climates across the west, with alpine conditions in the Snowy Mountains. Rainfall is highest along the coastal fringe and orographic zones, declining markedly inland, while temperature generally increases from the highlands to the western plains and during summer heatwaves. Hydro‐climatic variability is driven by large‐scale modes such as El Niño–Southern Oscillation and the Indian Ocean Dipole, with ex‐tropical systems and east‐coast lows periodically delivering intense rainfall to the north and central coast. Major river systems include coastal catchments (e.g., Hawkesbury–Nepean, Hunter, Richmond/Clarence) and the western Murray–Darling Basin (e.g., Murrumbidgee, Lachlan, Macquarie), creating flood‐prone floodplains in both coastal and inland LHDs. This strong spatial heterogeneity in baseline climate, extreme‐event exposure (heat, heavy rain, and flooding), settlement patterns, and infrastructure motivates small‐area analyses and provides a natural setting to examine how climate drivers translate into LHD‐specific *Salmonella* risk (Figure 1 top panel).

#### 2.1.1 Health Data

Monthly non‐identifiable notifications of *Salmonella* cases from 1991 to 2022 were obtained from the NSW Health Notifiable Conditions Information Management System (NCIMS), via the Communicable Diseases Branch and the Center for Epidemiology and Evidence, NSW Ministry of Health. These data were aggregated by LHD and used to calculate monthly incidence rates. LHD population data were sourced from the NSW Department of Planning (NSW Department of PlanningHousing and Infrastructure, 2024). For years before 2001, historical population estimates were retrospectively calculated using the department's earliest available annual growth rates (from 2002), applied separately for each LHD. See (Figure 1 bottom panel) for a map of NSW showing the distribution during the study period.

#### 2.1.2 Meteorological and Hydrological Data

Meteorological and hydrological data were extracted from the Australian Bureau of Meteorology at the weather station level, including mean temperature (°C), mean maximum temperature (°C), mean minimum temperature (°C), daily total rainfall (mm) and streamflow ($ML\;day^{-1}$; $1\;ML\;day^{-1}=1000\;m^{3}\;day^{-1}$) for the years 1991–2022. Monthly Flood wave events were defined as the number of days with river discharge/streamflow exceeding the 95th percentile of the distribution. As the meteorological records are georeferenced but not labeled by LHD, the coordinates of each weather station were manually assigned to the corresponding LHD using Google Maps. Where multiple stations existed within an LHD, values were averaged as appropriate.

### 2.2 Data Analysis

The preliminary analysis includes the annual *Salmonella* incidence rates per 100,000 population calculated for each LHD. To explore exposure‐response relationships, we produced scatter plots with regression lines to visualize the association between *Salmonella* incidence and climate variables, rainfall by LHD. In addition, autocorrelation and partial autocorrelation functions (ACF and PACF) were calculated to assess the temporal dependence on *the* incidence of Salmonella (Supporting Information S1).

#### 2.2.1 Climate–*Salmonella* Associations

**Figure 2:**
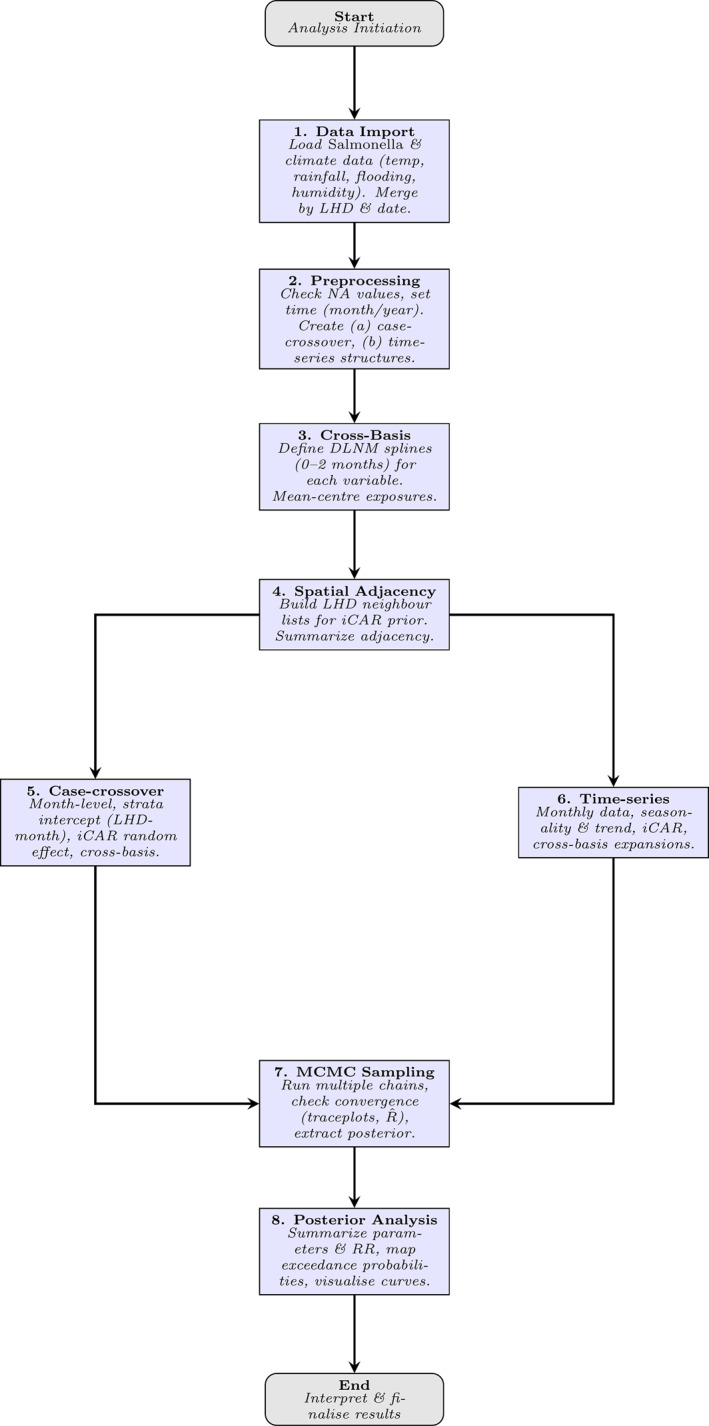
Methodological flowchart.

To investigate how climatic factors influence *Salmonella* incidence across NSW, we applied SB‐DLNMs, constructing distributed lag non‐linear terms for each climate exposure to capture potentially non‐linear and delayed associations flexibly (Gasparrini et al., 2010; Quijal‐Zamorano et al., 2024). The monthly *Salmonella* counts for each LHD were linked with the corresponding climate data, including mean temperature, total rainfall, a flooding index, and mean humidity. These data were structured into two formats to support complementary modeling strategies: a crossover case data set, where each month was nested within the LHD‐month strata (allowing within‐stratum comparisons) and a time‐series data set, indexed by year and month per LHD (Figure 2).

For each climatic exposure, we defined a cross‐basis using natural cubic splines to flexibly capture both the non‐linear exposure–response relationships and lagged effects up to 2 months. Exposure splines used knots in the 10th, 50th, and 90th percentiles of observed values, while lag splines spanned lags of 0–2 months. All exposures were mean‐centered prior to basis construction, and the resulting basis matrices were appended to the data sets to model both immediate and delayed risk. We report the relative risk (RR), a measure of association which denotes the ratio of expected incidence at exposure x to that at a reference $x_{\text{ref}}$, that is, $RR\left(x;x_{\text{ref}}\right)=\lambda \left(x\right)/\lambda \left(x_{\text{ref}}\right)=\exp\left\{\eta \left(x\right)-\eta \left(x_{\text{ref}}\right)\right\}$. We present percentile contrasts such as $RR_{99}$ comparing the 99th to the 5th percentile, representing cumulative effects over lags 0–2 months unless indicated otherwise. The 5th and 95th percentiles are used for primary inference as within‐sample extremes that avoid sparsity‐driven tail behavior; 1st and 99th percentile contrasts are shown only for completeness and are interpreted with explicit caveats. To reduce tail‐driven instability, boundary knots for exposure splines were set at the empirical 1st and 99th percentiles, and exposures were winsorized at [1st, 99th] prior to cross‐basis construction. This spatial Bayesian DLNM was chosen because climate–health associations are non‐linear, delayed, and spatially heterogeneous; it provides flexible exposure–lag curves with partial pooling across neighboring LHDs and coherent uncertainty (including exceedance probabilities) (Gasparrini et al., 2010; Lawson, 2018; Quijal‐Zamorano et al., 2024).

Monthly *Salmonella* case counts, $y_{i}$, were modeled using a Poisson likelihood, with the logarithm of the expected count, $\log\left(\lambda_{i}\right)$, serving as the linear predictor (Besag et al., 1991; Maclure, 1991). Two Bayesian models were fitted to quantify the associations between climatic variables and *Salmonella* risk, incorporating both spatial and temporal variability in the exposure‐response relationships.

First, a time‐stratified case‐crossover design (Maclure, 1991) was used, using LHD–month strata as fixed intercepts, inherently controlling for time‐varying confounders like seasonality and long‐term trends:

$$\log\left(\lambda_{i}\right)=\alpha_{\text{stratum}\left(i\right)}+\sum_{\text{exposures}}f_{r\left(i\right)}\left(X_{i}\right)+\phi_{r\left(i\right)},$$

where $\alpha_{\text{stratum}\left(i\right)}$ is the stratum‐specific intercept for the LHD × (month–year) stratum of observation i, $f_{r}\left(X_{i}\right)$ represents the DLNM basis contribution for each exposure X in region r, and $\phi_{r\left(i\right)}$ is the spatial random effect for the region of observation i. The long‐term trends and seasonality are handled implicitly via a time‐stratified case‐crossover design, where each LHD × month–year combination is treated as a fixed‐effect stratum $\alpha_{\text{stratum}\left(i\right)}$, absorbing time‐varying confounders without requiring explicit temporal terms.

Secondly, a full Bayesian hierarchical time‐series structure with random intercepts for LHD and calendar month was included, along with temporal random effects to capture region‐specific long‐term trends and anomalies. A spatially structured random effect $\left(\phi_{r}\right)$ was included using an iCAR prior to account for spatial correlation between neighboring LHDs (Besag et al., 1991). Region‐specific exposure–response curves were estimated using partial pooling around a global mean curve, with shrinkage determined by variance hyperparameters. This framework allowed estimation of both overall and LHD‐specific climate–*Salmonella* associations, accounting for spatial and temporal dependencies:

$$\log\left(\lambda_{i}\right)=\alpha_{r\left(i\right),\;m\left(i\right)}+\gamma_{r\left(i\right)}\left({\text{trend}}_{i}\right)+\delta_{r\left(i\right),\;y\left(i\right),\;s\left(i\right)}+\sum_{\text{exposures}}f_{r\left(i\right)}\left(X_{i}\right)+\phi_{r\left(i\right)},$$

where $\alpha_{r,m}$ is the intercept for region r in month m (capturing baseline seasonal variation), $\gamma_{r}\left(\text{trend}\right)$ is a region‐specific long‐term trend term (modeled with basis functions of time), and $\delta_{r,y,s}$ is a region‐ and year‐specific seasonal deviation (an adjustment for season s in year y). These terms allow the model to account for secular trends and inter‐annual variability in seasonal peaks.

To assess the incremental contribution of each exposure, we ran likelihood–ratio tests (LRTs) comparing the full DLNM to leave‐one‐out reduced models (removing one exposure cross‐basis at a time). Because counts showed overdispersion, deviance differences were scaled by $\hat{c}$ (Pearson $\chi^{2}$/residual df from the full time‐series model; $\hat{c}\approx 2.82$) and referenced to a $\chi^{2}$ distribution with degrees of freedom equal to the removed cross‐basis dimension. Table 1 lists the notation used in this paper.

**Table 1:** Notation Used

Symbol	Definition
i	Observation index (LHD–month)
r; m; y; s	Region (LHD); month; year; season
$y_{i}$	Observed monthly *Salmonella* count
$\lambda_{i}$	Expected count; linear predictor is $\log\left(\lambda_{i}\right)$
$X_{i}$	Climate exposures: temperature, rainfall, flooding, humidity
$f_{r}\left(X_{i}\right)$	Region‐specific DLNM cross‐basis contribution
$\alpha_{\text{stratum}\left(i\right)}$	CCM stratum intercept (LHD×month–year)
$\alpha_{r,m}$	Region–month intercept (baseline seasonality) in BSM
$\gamma_{r}\left({\text{trend}}_{i}\right)$	Region‐specific long‐term trend term
$\delta_{r,y,s}$	Region–year seasonal deviation
$\phi_{r}$	Spatial random effect (iCAR prior)

### 2.3 Bayesian Modeling Overview

We adopt a Bayesian hierarchical framework to estimate climate–*Salmonella* associations across LHDs. In this approach, uncertainty is propagated from data and parameters through prior distributions to posterior inference, allowing coherent quantification of both effect sizes and uncertainty at multiple levels (e.g., state‐wide and LHD‐specific) (Lawson, 2018). Let $y_{r,t}$ denote the monthly *Salmonella* count in LHD r and month t. We model $y_{r,t}∼Poisson\left(\lambda_{r,t}\right)$ with a log link for $\lambda_{r,t}$, and represent exposure–response functions using flexible bases so that non‐linear and lagged effects can be learned from the data while sharing information between districts via partial pooling.

Spatial dependence between neighboring LHDs is introduced through an iCAR prior on spatial random effects, encouraging similar baseline risks for adjacent regions while permitting localized deviations (Besag et al., 1991; Lawson, 2018). To capture non‐linear and delayed climate effects, we use a DLNM cross‐basis terms; these parameterize smooth responses over exposure and lag, and can be embedded naturally in the hierarchical structure (Gasparrini et al., 2010). Recent developments extend DLNMs to a SB‐DLNM, enabling joint estimation of area‐specific exposure–lag curves with spatial smoothing and borrowing of strength across small areas (Quijal‐Zamorano et al., 2024).

Inference proceeds by Markov chain Monte Carlo (MCMC) using NIMBLE in *R*, which provides a flexible platform for user‐defined hierarchical models and efficient sampling of large latent Gaussian structures (de Valpine et al., 2017). We summarize effects as RRs for percentile contrasts of each exposure, along with exceedance probabilities P(RR>τ) to support decision‐relevant thresholds. Model adequacy and parsimony are compared using the deviance information criterion (DIC) (Spiegelhalter et al., 2002); for completeness, we also report Bayesian information criterion (BIC) (Schwarz, 1978) and an overdispersion‐adjusted QAIC where appropriate (Burnham & Anderson, 2002). This framework allows us to quantify non‐linear, lagged, and spatially varying climate–disease relationships while providing uncertainty‐aware summaries useful for risk management.

### 2.4 Bayesian Model Specification, Priors, and Computation

For both models, priors for all regression coefficients in the exposure cross‐basis were given hierarchical normal distributions:

$$\beta_{r,j}^{\left(X\right)}=\mu_{j}^{\left(X\right)}+\sigma_{j}^{\left(X\right)}\;\theta_{r,j}^{\left(X\right)},\;\theta_{r,j}^{\left(X\right)}∼N\left(0,\tau_{X}^{-1}\right),$$

with vague hyperpriors such as

$$\mu_{j}^{\left(X\right)}∼N\left(0,10^{3}\right),\;\sigma_{j}^{\left(X\right)}∼U\left(0,10\right),\;\tau_{X}∼\text{Gamma}\left(0.1,0.1\right).$$

Intercepts and other random effects were given diffuse priors (e.g., $\alpha ∼N\left(0,10^{3}\right)$), and the iCAR precision $\tau_{\phi }$ also had a Gamma(0.1,0.1) prior. The models were fitted with three MCMC chains of 5,000 iterations each (after burn‐in), and convergence was assessed by traceplots and $\hat{R}$ statistics. Posterior samples of all key parameters (including derived quantities such as RRs) were extracted for inference.

All models were fitted in a Bayesian framework using the NIMBLE package (de Valpine et al., 2017) in *R* version 4.3.3.

## 3 Results

### 3.1 Model Comparison

A comparison between the time‐stratified case‐crossover model (CCM) and the full Bayesian spatiotemporal model (BSM) is provided in Table S1 in Supporting Information S1. Across nearly all performance metrics, BSM demonstrated superior model fit and predictive accuracy. Specifically, it yielded lower root mean squared error and mean absolute error in case predictions, and a higher pseudo‐$R^{2}$ (0.854 vs. 0.827), indicating greater explanatory power. Model selection criteria also favored BSM, with markedly lower values for DIC, Akaike information criterion, and BIC. Although CCM showed marginally better performance based on quasi‐AIC (QAIC) derived from an overdispersion‐adjusted likelihood, the Bayesian QAIC from posterior deviance supported BSM. Given BSM's enhanced ability to account for seasonality and long‐term trends, all subsequent results presented in this main manuscript are based on the BSM. Results from CCM models are presented in the Supporting Information S1.

Across all three criteria, the time‐series design model is preferred. Its DIC is lower by about 213, indicating better expected out‐of‐sample fit after penalizing effective complexity (Spiegelhalter et al., 2002). The BIC difference is large (about 27,430), which strongly favors the time‐series design under BIC's heavier penalty on parameters (Schwarz, 1978). The overdispersion‐adjusted criterion (QAIC) is also notably smaller (7,978 vs. 11,545), supporting superior predictive adequacy when extra‐Poisson variation is present (Burnham & Anderson, 2002). Taken together, these diagnostics justify using the time‐series design model for primary inference. Model‐selection diagnostics (DIC, BIC, QAIC) are summarized in Table 2, which favors the time‐series design model over the case‐crossover design.

**Table 2:** Model‐Selection Diagnostics for Competing Specifications (Lower Is Better)

Specification	DIC	BIC	QAIC
Case‐crossover design	33,710.56	77,916.64	11,545.03
Time‐series design model	33,497.14	50,486.43	7,978.30

### 3.2 Exploratory Spatial Data Analysis

**Figure 3:**
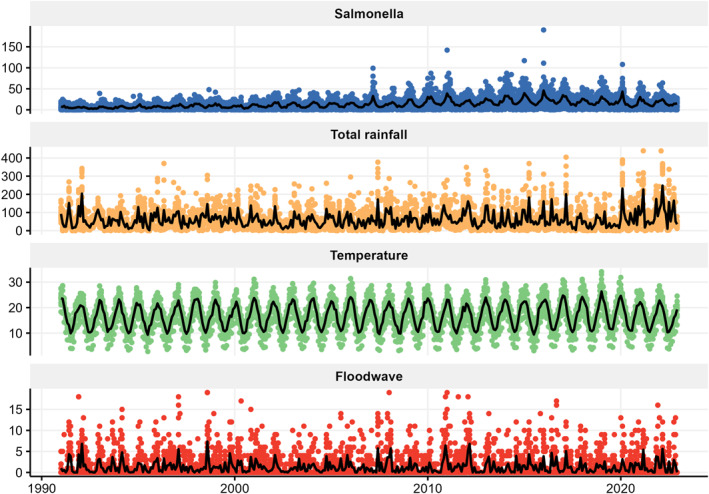
Time series plot of *Salmonella* cases, total rainfall, mean temperature and flood waves over the study period, 1991–2022. Thick lines represent monthly averages, while each point represents values for each Local Health District.

Between 1991 and 2022, the mean monthly number of *Salmonella* cases was 13.3 (SD = 13.3), with a median of 9 cases. The maximum monthly count was 190 cases, recorded in South Eastern Sydney in January 2016, while none were recorded in NSW during the 1990s (Table 3). Figure 3 shows peaks of *Salmonella* cases recorded during the years 2007–2008, 2010–2012, 2014–2016 and 2020–2021. Increased flood wave events were recorded over 2007, 2011–2013, 2016–2017 and 2020–2022. These are in keeping with recorded major flooding events in NSW, such as the Hunter Valley and Central Coast floods in 2007, Wollongong floods in 2011, Northern NSW floods in 2012–2013, Central West and Riverina floods in 2016, Cyclone Debbie in 2017 and NSW North Coast floods in 2020–2021. The figure also illustrates numerous flood wave events recorded during the 1990s, which coincide with increased *Salmonella* case recordings; however, these rises are not as pronounced as those seen in the 2000s.

**Table 3:** Summary of Monthly *Salmonella* Cases and Climate Variables for New South Wales Local Health Districts, 1991–2022

Variable	Mean	SD	Min	25th	Median	75th	99th	Max
1. Max. Temp	21.51	5.34	7.15	17.5	21.46	25.1	34.5	40.2
2. Mean Temp	16.75	5.09	2.75	12.9	16.92	20.7	28.1	34.1
3. Min. Temp	12.53	5.08	−0.6	8.52	12.7	16.7	21.9	27.9
4. Rainfall (mm)	60.66	55.7	0	0	0	2	275.8	493
5. Flood Events	1.25	2.53	0	0	0	2	12	19
6. *Salmonella* Cases	13.33	13.3	0	4	9	18	60	190

*Note.* Statistics are over all LHD–month observations. Temperature is monthly mean (°C); rainfall is monthly total (mm; daily sums, station‐averaged by LHD); flood events are days per month with streamflow.

Time series decomposition of *Salmonella* cases in NSW reveals a gradual upward trend, particularly between 2000 and 2015, and a consistent seasonal pattern with annual peaks (Figure S1 in Supporting Information S1). Irregular spikes in the residual component suggest sporadic outbreaks. Associations between monthly mean temperature and *Salmonella* cases across 15 LHDs (Figure S2 in Supporting Information S1), and total monthly rainfall (Figure S3 in Supporting Information S1), indicate that temperature has the strongest association.

### 3.3 Spatial Temporal Association

**Figure 4:**
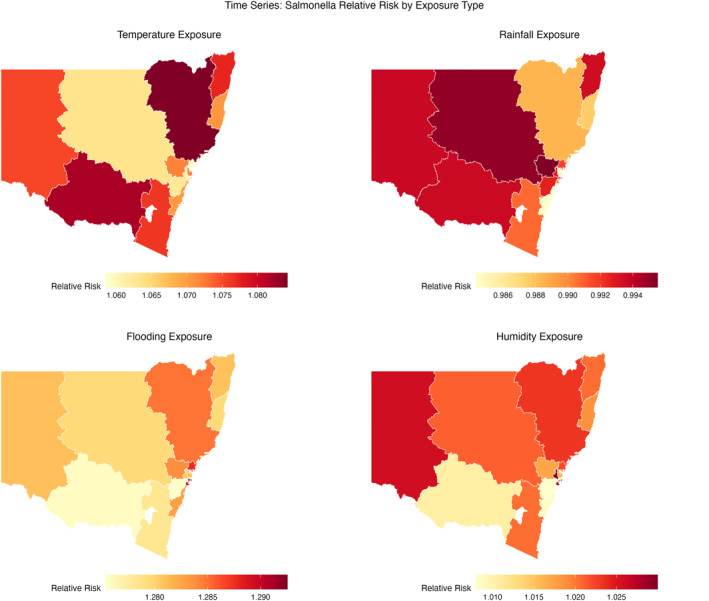
Spatial distribution of the estimated relative risk (RR) of *Salmonella* associated with extreme values of each climate variable under Bayesian spatiotemporal model. Panels show posterior median RR by Local Health District (LHD) for: Temperature (hot vs. cool conditions), Rainfall (wet vs. dry conditions), Flooding (occurrence of a flood‐wave vs. none), and Humidity (high vs. low). Darker colors indicate higher risk. Temperature and humidity display positive associations across most of NSW; heavy rainfall is generally protective (lighter shades), whereas flooding shows a strong positive association across many LHDs.

**Figure 5:**
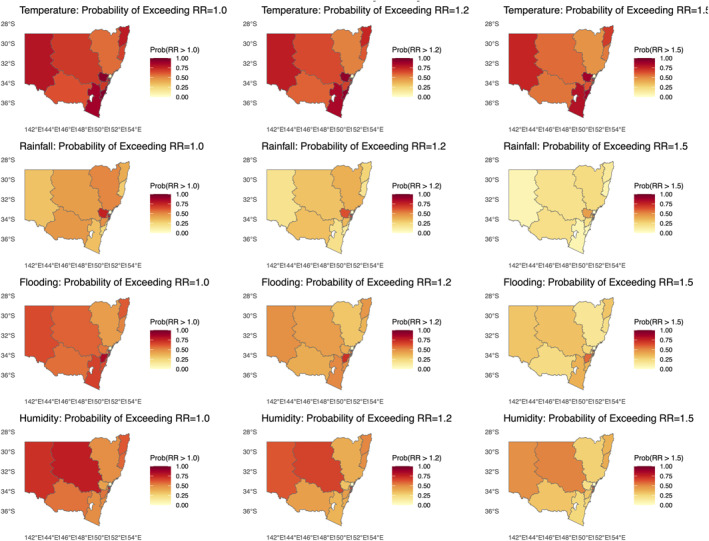
Posterior probability that the relative risk (RR) exceeds thresholds (1.0, 1.2, 1.5) for each exposure in Bayesian spatiotemporal model. Shading indicates the probability level in each Local Health District.

Figure 4 and Table 4 show apparent spatial heterogeneity in climate–*Salmonella* associations. Temperature is positive across all LHDs: moving from the fifth to the 99th percentile yields RRs typically 2.4–4.8 (largest in Hunter, New England). Rainfall exhibits the opposite direction: dry months (1st–5th percentile) are associated with higher risk (RR>1), while very wet months (95th–99th) are generally protective (RR<1) in all LHD, strongest in coastal districts. Flooding shows a strong positive association throughout the state, with ${\text{RR}}_{95}\approx$ 5.5–6.3 and ${\text{RR}}_{99}\approx$ 18–23.5 (largest in Sydney). Humidity effects are smaller but consistently positive (${\text{RR}}_{99}\approx$ 1.1–1.5), with higher values in Western and South Eastern Sydney. We also mapped the posterior probability that RR exceeds thresholds such as 1.0, 1.2, and 1.5 (Figure 5). These maps show high P(RR>1) for temperature and flooding across most LHDs and, conversely, high P(RR<1) for heavy rainfall, with localized variation by district.

**Table 4:** Percentile‐Based Relative Risk (RR) of *Salmonella* by Local Health District and Climate Variable (Model4̃)

LHD_Region	Exposure_Variable	Percentile_01	Percentile_05	Percentile_95	Percentile_99
Central Coast	Temperature	0.767	0.941	2.105	2.508
Central Coast	Rainfall	1.465	1.424	0.363	0.150
Central Coast	Flooding	1.000	1.000	5.870	20.781
Central Coast	Humidity	0.406	0.522	1.252	1.329
Far West	Temperature	0.683	0.916	2.916	3.752
Far West	Rainfall	1.341	1.311	0.460	0.233
Far West	Flooding	1.000	1.000	5.664	19.546
Far West	Humidity	0.348	0.467	1.300	1.395
Hunter New England	Temperature	0.638	0.902	3.539	4.766
Hunter New England	Rainfall	1.713	1.645	0.240	0.069
Hunter New England	Flooding	1.000	1.000	5.782	20.250
Hunter New England	Humidity	0.386	0.504	1.267	1.350
Illawarra Shoalhaven	Temperature	0.700	0.921	2.726	3.452
Illawarra Shoalhaven	Rainfall	2.233	2.101	0.119	0.019
Illawarra Shoalhaven	Flooding	1.000	1.000	5.717	19.861
Illawarra Shoalhaven	Humidity	0.705	0.777	1.091	1.117
Mid North Coast	Temperature	0.699	0.921	2.736	3.467
Mid North Coast	Rainfall	1.779	1.703	0.217	0.057
Mid North Coast	Flooding	1.000	1.000	5.614	19.252
Mid North Coast	Humidity	0.466	0.577	1.209	1.272
Murrumbidgee	Temperature	0.661	0.909	3.201	4.211
Murrumbidgee	Rainfall	1.342	1.312	0.459	0.232
Murrumbidgee	Flooding	1.000	1.000	5.504	18.611
Murrumbidgee	Humidity	0.650	0.733	1.113	1.145
Nepean Blue Mountains	Temperature	0.694	0.919	2.789	3.552
Nepean Blue Mountains	Rainfall	1.172	1.158	0.657	0.456
Nepean Blue Mountains	Flooding	1.000	1.000	5.748	20.045
Nepean Blue Mountains	Humidity	0.494	0.602	1.192	1.249
Northern NSW	Temperature	0.676	0.914	3.001	3.888
Northern NSW	Rainfall	1.353	1.322	0.449	0.223
Northern NSW	Flooding	1.000	1.000	5.660	19.524
Northern NSW	Humidity	0.432	0.546	1.232	1.303
Northern Sydney	Temperature	0.692	0.919	2.810	3.584
Northern Sydney	Rainfall	2.009	1.905	0.158	0.031
Northern Sydney	Flooding	1.000	1.000	5.666	19.560
Northern Sydney	Humidity	0.511	0.616	1.182	1.236
South Eastern Sydney	Temperature	0.706	0.923	2.662	3.353
South Eastern Sydney	Rainfall	1.573	1.520	0.301	0.105
South Eastern Sydney	Flooding	1.000	1.000	5.910	21.022
South Eastern Sydney	Humidity	0.305	0.425	1.343	1.454
South Western Sydney	Temperature	0.727	0.929	2.448	3.023
South Western Sydney	Rainfall	1.406	1.370	0.406	0.184
South Western Sydney	Flooding	1.000	1.000	5.453	18.312
South Western Sydney	Humidity	0.757	0.818	1.072	1.092
Southern NSW	Temperature	0.680	0.915	2.961	3.824
Southern NSW	Rainfall	1.532	1.484	0.323	0.120
Southern NSW	Flooding	1.000	1.000	5.577	19.037
Southern NSW	Humidity	0.433	0.547	1.232	1.302
Sydney	Temperature	0.731	0.930	2.411	2.966
Sydney	Rainfall	1.377	1.344	0.429	0.204
Sydney	Flooding	1.000	1.000	6.306	23.497
Sydney	Humidity	0.623	0.711	1.125	1.161
Western NSW	Temperature	0.725	0.929	2.469	3.055
Western NSW	Rainfall	1.256	1.234	0.547	0.323
Western NSW	Flooding	1.000	1.000	5.615	19.256
Western NSW	Humidity	0.423	0.538	1.239	1.312
Western Sydney	Temperature	0.729	0.930	2.427	2.991
Western Sydney	Rainfall	1.399	1.364	0.411	0.189
Western Sydney	Flooding	1.000	1.000	5.652	19.475
Western Sydney	Humidity	0.272	0.391	1.382	1.508

Table 4 indicates non‐linear behavior that differs by exposure. For temperature and humidity, associations are J‐shaped: low percentiles are at or below 1.0, while high percentiles exceed 1.0 (temperature up to ∼4.8; humidity up to ∼1.5). By contrast, rainfall shows an inverse pattern: dry conditions elevate risk (RR 1.17–2.23 at the 1st–5th percentiles), whereas very wet conditions are generally protective (${\text{RR}}_{99}$ 0.02–0.66, varying by LHD). The flooding index is monotone positive, with sharp increases at high percentiles (${\text{RR}}_{95}\approx$ 5.5–6.3; ${\text{RR}}_{99}\approx$ 18–23.5), largest in Sydney (${\text{RR}}_{99}$ = 23.5) and South Eastern Sydney (21.0). Illustratively, the Murrumbidgee LHD shows rainfall RR decreasing from 1.34 at the 1st percentile to 0.23 at the 99th percentile, indicating substantially *lower* risk in very wet months than in very dry months. Temperature effects are positive across all LHDs—for example, Hunter New England reaches ${\text{RR}}_{99}$ = 4.77—while humidity effects are smaller but consistently above 1 at high percentiles (e.g., South Eastern Sydney 1.45 vs. 0.31 at the 1st percentile). Overall, these patterns suggest heat and sustained moisture favor transmission (with humidity adding modest increments), background rainfall may dilute/disrupt exposure pathways, and floods likely mobilize contamination and overwhelm infrastructure. Effect sizes vary by LHD: inland/rural districts tend to show larger heat contrasts, while dense coastal/metropolitan systems exhibit the strongest flood sensitivity.

### 3.4 Variable Importance

**Figure 6:**
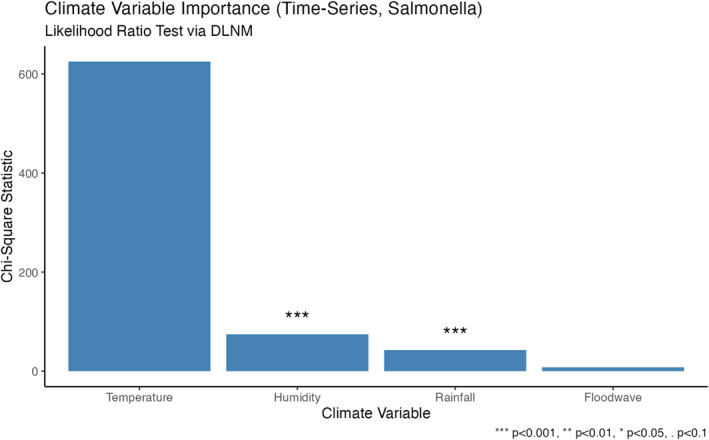
Variable importance from likelihood–ratio tests (full time‐series Distributed lag non‐linear model vs. leave‐one‐out reduced models). Bars show the $\chi^{2}$ statistics derived from ΔDeviance with degrees of freedom equal to the removed cross‐basis dimensions (temperature, humidity, rainfall: 12 df; floodwave: 6 df). Significance codes: ***p<0.001, **p<0.01, *p<0.05, 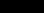.

Likelihood–ratio tests indicate that temperature is the dominant driver of variability in salmonellosis (scaled ΔDeviance $\chi_{12}^{2}\approx 624.9$, p<0.0001), with smaller but statistically significant contributions from humidity ($\chi_{12}^{2}\approx 74.4$, p<0.0001) and rainfall ($\chi_{12}^{2}\approx 42.7$, p<0.0001). Flood wave did not improve overall fit once other exposures were included ($\chi_{6}^{2}\approx 7.83$, p=0.25). This ranking aligns with the spatial RR maps and exceedance probabilities, where heat effects are strongest statewide and humidity/rainfall adds a modest signal (Figure 6).

## 4 Discussion

Our analyses highlight the significant influence of meteorological factors, specifically temperature, humidity, and, under certain conditions, rainfall and flooding, on the incidence of *Salmonella* in NSW. By employing two complementary Bayesian modeling approaches, a case‐crossover design and a spatiotemporal time‐series framework, this dual approach allowed for robust validation of climate‐disease associations and underscored the importance of accounting for seasonality, long‐term trends, and localized spatial heterogeneity in epidemiological modeling.

### 4.1 Non‐Linear Relationships and Extreme Percentiles

A principal finding is the markedly non‐linear association between climatic exposures and *Salmonella* risk. Percentile contrasts (1st, 5th, 95th, 99th) reveal pronounced U/J‐shaped curves across most LHDs: at lower percentiles (cooler or drier conditions), RRs frequently fall below one. In comparison, at higher percentiles—especially beyond the 95th‐risks rise sharply. Several LHDs exhibit sizable increases at extremes; for example, the Far West shows high heat‐related RRs, and Illawarra Shoal haven displays large surges at the upper flood‐index tail. These patterns underscore the potential for rapid escalation of *Salmonella* risk during heatwaves, intense rainfall, flooding, or unusually humid periods. The shapes are consistent with laboratory and environmental evidence: *Salmonella* growth accelerates above thermal thresholds, and precipitation can mobilize environmental pathogens (D’Souza et al., 2004; Baker et al., 2019). This behavior aligns with observed seasonality and survival dynamics. Infections tend to decline during cold spells and increase during hot weather (D’Souza et al., 2004), and hot conditions are a well‐established driver of outbreaks (Scallan et al., 2011). Pooled analyses suggest an approximately 5% increase in infection per 1°C of warming (Williams et al., 2020), implying that at very high percentiles, small additional temperature increases can compound into large RRs. Even brief heat waves can substantially increase risk (Manchal et al., 2024, 2025; Milazzo et al., 2016b). In Australia, temperatures above 41°C were associated with a 34% increase in salmonellosis (Milazzo et al., 2016b). Overall, the temperature–*Salmonella* relationship appears strongly non‐linear, with risk accelerating once critical heat and moisture thresholds are exceeded (D’Souza et al., 2004; Baker et al., 2019; Scallan et al., 2011; Williams et al., 2020; Milazzo et al., 2016b; Zhang et al., 2010). For context, pooled epidemiological analyses suggest approximately a 5%–10% increase in salmonellosis per 1°C warming, and an Australian study reported a 34% increase during days exceeding 41°C (Milazzo et al., 2016b; Williams et al., 2020). The magnitude of our 5th–95th temperature contrasts aligns with this literature. In a few LHDs, 99th‐percentile contrasts produced very large RRs with wide credible intervals. These arise from sparse support at the extreme tails and spline curvature, and should not be interpreted as precise magnitudes. Consistent with prior epidemiological evidence, effect sizes within the well‐supported 5th–95th percentile range align with gradual increases of approximately 5% per 1°C and about 34% during brief extreme heat events (Milazzo et al., 2016b; Williams et al., 2020).

#### 4.1.1 Probability of Exceeding Thresholds

Complementing the point estimates, exceedance–probability maps provide a clear view of where and with what certainty climate effects are likely to breach operational thresholds, summarizing both magnitude and uncertainty in a single surface (Gasparrini et al., 2010; Lawson, 2018; Quijal‐Zamorano et al., 2024). In our results, large parts of NSW show high probability (P>0.7) that risk increases under extreme heat or high humidity, consistent with the positive temperature and moisture responses seen in the percentile analyses. By contrast, for heavy rainfall the maps frequently indicate P(RR>1)<0.5, reflecting the generally protective signal of very wet months at the aggregated monthly scale. Spatial variation is evident: inland and semi‐rural LHDs often register higher heat‐related exceedance probabilities, while some metropolitan/coastal LHDs exhibit high probabilities for flood‐related increases, consistent with contamination pathways during high‐flow events and infrastructure stress (Ebi et al., 2006; Tan et al., 2004). Overlaying these probabilities on specific regions helps identify potential hotspots for action. For example, western Sydney shows non‐trivial probabilities of flood‐driven surges, whereas the Far West displays higher probabilities of exceeding heat thresholds than flood thresholds, aligning with its arid baseline climate. Framing results as P(RR>τ) for thresholds such as 1.0, 1.2, or 1.5 enables risk managers to calibrate responses to their tolerance for false positives/negatives and to trigger targeted interventions when forecasts indicate a high chance of exceedance (Lawson, 2018; Quijal‐Zamorano et al., 2024). In practice, these maps can guide the pre‐deployment of food safety inspections, intensified water quality monitoring, and tailored public advisories ahead of heatwaves and flood forecasts, while communicating uncertainty transparently to stakeholders (Ebi et al., 2006; Tan et al., 2004).

### 4.2 Climate Specifics

Consistently across all LHDs, elevated ambient temperature was associated with higher *Salmonella* risk, in line with temperature‐dependent growth and potential strain on cold chains (typical ${\text{RR}}_{99}$ about 2.4–4.8) (D’Souza et al., 2004; Scallan et al., 2011; Williams et al., 2020; Milazzo et al., 2016b). High humidity added a smaller increment to risk, plausibly by prolonging environmental survival (${\text{RR}}_{99}$ about 1.1–1.5) (Baker et al., 2019). By contrast, monthly rainfall showed a more complex pattern: very wet months were generally protective (${\text{RR}}_{99}<$1), consistent with dilution, behavioral changes, or the month‐aggregation structure (Curriero et al., 2001). However, discrete flood events, distinct from monthly totals, were strongly and positively associated with risk, plausibly via sewer or stormwater overflow and contamination pathways (Ebi et al., 2006; Tan et al., 2004). This combination helps explain why some inland or semi‐rural LHDs (e.g., Murrumbidgee, Western Sydney) can exhibit marked increases during high‐flow episodes even when background rainfall appears neutral or protective (Ebi et al., 2006; Tan et al., 2004).

Flood‐linked risks showed pronounced spatial heterogeneity, with certain LHDs (e.g., Illawarra Shoalhaven, South Western Sydney) experiencing substantial surges at the highest flood index percentiles. These patterns are plausibly driven by flood‐related disruption of critical infrastructure such as sewage and water treatment, leading to wider environmental contamination. In contrast, negligible flood impacts in some districts may reflect more resilient systems or fewer high‐flow events. Elevated humidity was also positively associated with risk, though generally to a lesser degree than extreme temperature or flooding. Notably, a subset of inland districts (Far West, Western NSW) displayed marked increases at high humidity percentiles, consistent with the role of atmospheric moisture in sustaining bacterial persistence, particularly under hot conditions (Baker et al., 2019).

Mechanistically, high humidity can prolong the survival of *Salmonella* on foods and fomites by reducing desiccation, thereby amplifying heat effects (Baker et al., 2019). Conversely, dry periods tend to suppress transmission, whereas intense rainfall elevates it in line with the concentration–dilution hypothesis (Curriero et al., 2001). During extended dry spells, environmental pathogen loads may fall; heavy rainfall can then wash organisms from soil or animal waste into water sources and food‐production environments, increasing exposure risk (Tan et al., 2004).

### 4.3 Spatial Heterogeneity and Local Vulnerabilities

A key insight from this study is the substantial spatial heterogeneity in climate sensitivity observed across the 15 LHDs. Effect sizes vary by LHD. Temperature effects peak in Hunter New England (${\text{RR}}_{99}$ about 4.77) and are otherwise about 2.4–4.0 across the state. Flooding effects are strongest in some metropolitan or coastal LHDs (e.g., Sydney, about 23.5; South Eastern Sydney, about 21.0), indicating vulnerability of dense stormwater or sewer networks. Humidity effects are modest but higher in Western and South Eastern Sydney (about 1.4–1.5). Rainfall protection is strongest in coastal LHDs and weaker inland, yet ${\text{RR}}_{99}$¡1 statewide. These patterns suggest tailoring adaptation: heatwave alerts and cold‐chain reinforcement broadly, and flood‐readiness and wastewater resilience in high‐risk LHDs (Ferrand, 2014; NSW Government, 2024; Schramm et al., 2020).

### 4.4 Implications for Surveillance and Intervention

These findings carry significant implications for public health surveillance and intervention, particularly in the context of projected increases in the frequency and intensity of extreme weather events under climate change (Semenza & Menne, 2012). Our results underscore the potential for increased frequency and severity of *Salmonella* outbreaks in susceptible regions. Public health agencies can leverage these findings by integrating climate metrics into disease early‐warning systems, enabling the issuance of targeted alerts when forecasts indicate extreme heat or rainfall. This proactive approach allows the preemptive deployment of targeted interventions, including enhanced food safety inspections, rigorous water quality monitoring, and timely public advisories. Strengthening critical infrastructure, such as sewer systems and storm water drainage, and ensuring reliable refrigeration capacity are especially vital in regions identified as highly vulnerable. Furthermore, public health education campaigns promoting safe food handling practices during heatwaves or floods (e.g., preventing cross‐contamination, recommending boiling water in post‐flood scenarios) can play a crucial role in mitigating the severity of outbreaks (World Health Organization, 2018a). Ultimately, the development and implementation of regional adaptation strategies that specifically address the localized climate risks identified in this study, such as extreme heat in the Far West, flooding vulnerabilities in Illawarra, and combined risks in areas like Western Sydney, are likely to be the most effective.

### 4.5 Strengths and Limitations

One of the main strengths of this study is that it leverages a long‐term data set to explore the relationship between environmental exposures and *Salmonella* risks in multiple LHDs in NSW. The inclusion of distributed lag structures and spatial disaggregation enables a robust investigation of temporal and regional variations in risk.

We acknowledge the following limitations in this study. First, residual confounding by unmeasured variables such as air pollution, socioeconomic status, or individual‐level comorbidities cannot be excluded. The reliance on aggregated data limits the ability to examine effect modification by demographic factors (e.g., age, sex) or behavioral exposures. Second, the analysis does not distinguish between sporadic and outbreak‐associated cases, which can confound estimates of climate sensitivity, particularly during extreme weather events. Third, potential compound events were not explicitly modeled, limiting the insight into more complex exposure scenarios. Fourth, we did not explore how serotype‐specific data may constrain understanding of transmission dynamics across different strains of *Salmonella* under climate stress. Lastly, estimates at the highest percentiles can be inflated by spline curvature and sparse support. We therefore restricted primary inference to within‐data contrasts (5th–95th), winsorized exposures at [1st, 99th] when constructing the cross‐basis, and present 99th‐percentile contrasts only with explicit caution about uncertainty.

## 5 Conclusion

This study provides evidence that high ambient temperatures and elevated humidity levels are significant drivers of increased *Salmonella* risk in NSW, with effects manifesting over short lags. Although high rainfall was often associated with a reduced risk in many areas, particularly in coastal and temperate regions, suggesting a disruption of transmission pathways, extreme rain and flood events showed a more complex or modest potential to raise risk, albeit with notable spatial variability and associated uncertainty. In general, temperature and humidity emerge as key meteorological factors associated with an increased risk of *Salmonella*, showing regionally varying effects and short‐term associations. Understanding this interplay between climate and disease is essential for developing effective public health strategies and adaptation measures to protect food safety and prevent future *Salmonella* outbreaks in a changing climate.

## Ethics Statement

No specific ethics approval was required for this study as data analysis was based on publicly available data. *Salmonella* data were obtained from de‐identified notifiable disease data, whilst the climate data were obtained from the Australian Bureau of Meteorology.

## Conflict of Interest

The authors declare no conflicts of interest relevant to this study.

## Supporting information

Supporting Information S1

## Data Availability

*Salmonella* case data were obtained from the NSW Health NCIMS, through the Communicable Diseases Branch and the Centre for Epidemiology and Evidence, NSW Ministry of Health. The data sets and associated *R* codes are available on GitHub (https://github.com/oyeadegboye/Salmonella‐data‐repo and Zenodo (Adegboye & Khan, 2025). *R* version 4.3.3 was used to perform all data analysis and visualization presented in this study.

## References

[gh270085-bib-0001] Adegboye, O. , & Khan, M. A. (2025). oyeadegboye/salmonella‐data‐repo: Climate‐driven salmonella risk (0.1.0) [Dataset]. Zenodo. 10.5281/zenodo.17766458

[gh270085-bib-0002] Akil, L. , Ahmad, H. A. , & Reddy, R. S. (2014). Effects of climate change on salmonella infections. Foodborne Pathogens and Disease, 11(12), 974–980. PMID: 25496072. 10.1089/fpd.2014.1802 25496072 PMC4346543

[gh270085-bib-0003] Australian Government Department of Health and Aged Care . (2024). National notifiable diseases surveillance system (nndss) public dataset – Salmonella. (Dataset). Australian Government Department of Health and Aged Care. Retrieved from https://www.health.gov.au/resources/publications/national‐notifiable‐diseases‐surveillance‐system‐nndss‐public‐dataset‐salmonella?language=en

[gh270085-bib-0004] Baker, J. R. , Hardwick, L. , & Lane, D. W. (2019). Environmental conditions impacting survival of salmonella on surfaces. Applied and Environmental Microbiology, 85(6), e03124–18.

[gh270085-bib-0005] Besag, J. , York, J. , & Mollié, A. (1991). Bayesian image restoration, with two applications in spatial statistics. Annals of the Institute of Statistical Mathematics, 43(1), 1–59. 10.1007/bf00116466

[gh270085-bib-0006] Burnham, K. P. , & Anderson, D. R. (2002). Model selection and multimodel inference: A practical information‐theoretic approach (2nd ed.). Springer.

[gh270085-bib-0007] Curriero, F. C. , Patz, J. A. , Rose, J. B. , & Subhash, L. (2001). Temporal and environmental patterns of cryptosporidiosis and giardiasis in the chesapeake Bay region. American Journal of Epidemiology, 156(3), 288–296.

[gh270085-bib-0008] Darby, J. , & Sheorey, H. (2008). Searching for salmonella. Australian Journal of General Practice, 37(10), 806–810. 19002298

[gh270085-bib-0009] Davis, B. P. , Amin, J. , Graham, P. L. , & Beggs, P. J. (2022). Climate variability and change are drivers of salmonellosis in Australia: 1991 to 2019. Science of The Total Environment, 843, 156980. 10.1016/j.scitotenv.2022.156980 35764154

[gh270085-bib-0010] de Valpine, P. , Turek, D. , Paciorek, C. , Anderson‐Bergman, C. , Temple Lang, D. , & Bodik, R. (2017). Programming with models: Writing statistical algorithms for general model structures with nimble. Journal of Computational & Graphical Statistics, 26(2), 403–413. 10.1080/10618600.2016.1172487

[gh270085-bib-0011] D’Souza, R. M. , Becker, N. G. , Hall, G. , & Moodie, K. B. (2004). Does ambient temperature affect foodborne disease? Epidemiology, 15(1), 86–92. 10.1097/01.ede.0000101021.03453.3e 14712151

[gh270085-bib-0012] Ebi, K. L. , Burton, I. , & McGregor, G. R. (2006). Flooding and public health: A critical review of the evidence. Health Effects of Flooding, 367, 1183–1189.

[gh270085-bib-0013] Ferrand, E. A. (2014). Rainwater harvesting as an effective climate change adaptation strategy in rural and urban settings. In Managing water resources under climate uncertainty: Examples from Asia, Europe, latin america, and Australia (pp. 405–420). Springer.

[gh270085-bib-0014] Ford, L. , Glass, K. , Veitch, M. , Wardell, R. , Polkinghorne, B. , Dobbins, T. , et al. (2016). Increasing incidence of salmonella in Australia, 2000–2013. PLoS One, 11(10), e0163989. 10.1371/journal.pone.0163989 27732615 PMC5061413

[gh270085-bib-0015] Gasparrini, A. , Armstrong, B. , & Kenward, M. G. (2010). Distributed lag non‐linear models. Statistics in Medicine, 29(21), 2224–2234. 10.1002/sim.3940 20812303 PMC2998707

[gh270085-bib-0016] Ikuta, K. S. , Swetschinski, L. R. , Robles Aguilar, G. , Sharara, F. , Mestrovic, T. , Gray, A. P. , et al. (2022). Global mortality associated with 33 bacterial pathogens in 2019: A systematic analysis for the global burden of disease study 2019. The Lancet, 400(10369), 2221–2248. 10.1016/S0140-6736(22)02185-7 PMC9763654 36423648

[gh270085-bib-0017] Lawson, A. B. (2018). Bayesian disease mapping: Hierarchical modeling in spatial epidemiology (3rd ed.). Chapman and Hall/CRC. 10.1201/9781351271769

[gh270085-bib-0018] Maclure, M. (1991). The case‐crossover design: A method for studying transient effects on the risk of acute events. American Journal of Epidemiology, 133(2), 144–153. 10.1093/oxfordjournals.aje.a115853 1985444

[gh270085-bib-0019] Manchal, N. , Sumana, M. N. , Young, M. K. , Castellanos, M. E. , Leggat, P. , & Adegboye, O. A. (2025). The impact of meteorological variables on Salmonella bacteraemia in mysuru district, Karnataka state, India: A retrospective time‐series analysis. In Therapeutic advances in infectious diseases. 10.1177/20499361251389056 PMC12575929 41180260

[gh270085-bib-0020] Manchal, N. , Young, M. K. , Castellanos, M. E. , Leggat, P. , & Adegboye, O. (2024). A systematic review and meta‐analysis of ambient temperature and precipitation with infections from five food‐borne bacterial pathogens. Epidemiology and Infection, 152, e98. 10.1017/s0950268824000839 39168633 PMC11736460

[gh270085-bib-0021] Milazzo, A. , Giles, L. , Zhang, Y. , Koehler, A. , Hiller, J. , & Bi, P. (2016a). The effect of temperature on different salmonella serotypes during warm seasons in a mediterranean climate city, Adelaide, Australia. Epidemiology and Infection, 144(6), 1231–1240. 10.1017/s0950268815002587 26522685

[gh270085-bib-0022] Milazzo, A. , Giles, L. C. , Zhang, Y. , Koehler, A. P. , Hiller, J. E. , & Bi, P. (2016b). Heatwaves differentially affect risk of salmonella serotypes. Journal of Infection, 73(3), 231–240. 10.1016/j.jinf.2016.04.034 27317378

[gh270085-bib-0023] Mjiang, C. , Shaw, K. S. , Upperman, C. R. , Blythe, D. , Mitchell, C. , Murtugudde, R. , et al. (2021). Climate change, extreme events, and increased risk of salmonellosis: Foodborne diseases active surveillance network (foodnet), 2004–2014. Environmental Health, 20(1), 105. 10.1186/s12940-021-00787-y 34537076 PMC8449873

[gh270085-bib-0024] NSW Health . (2024). Salmonella fact sheet. Retrieved from https://www.health.nsw.gov.au/Infectious/factsheets/Pages/salmonella.aspx (Accessed: 2025‐04‐09)

[gh270085-bib-0025] NSW Department of Planning, Housing and Infrastructure . (2024). Population projections: Explore the data. Retrieved from https://www.planning.nsw.gov.au/data‐and‐insights/population‐projections/explore‐the‐data

[gh270085-bib-0026] NSW Government . (2024). Nsw climate change adaptation action plan 2025–2029. Retrieved from https://www.climatechange.environment.nsw.gov.au/about‐adaptnsw/nsw‐government‐action‐climate‐change/Adaptation‐Action‐Plan‐2025‐2029. Accessed 26 06 2025.

[gh270085-bib-0039] NSW Treasury . (2021). Intergenerational report treasury technical research paper series: An indicative assessment of four key areas of climate risk for the 2021 NSW intergenerational report. Retrieved from https://www.treasury.nsw.gov.au/nsw‐economy/nsw‐intergenerational‐report/nsw‐intergenerational‐report‐treasury‐technical‐research

[gh270085-bib-0027] Quijal‐Zamorano, M. , Martinez‐Beneito, M. A. , Ballester, J. , & Marí‐Dell’Olmo, M. (2024). Spatial bayesian distributed lag non‐linear models (sb‐dlnm) for small‐area exposure‐lag‐response epidemiological modelling. International Journal of Epidemiology, 53(3), dyae061. 10.1093/ije/dyae061 38641428 PMC11031409

[gh270085-bib-0028] Ratnayake, H. , Eisen, D. , Adegboye, O. , Pak, A. , & McBryde, E. (2024). Bacteraemia in tropical Australia: A review. Current Tropical Medicine Reports, 11(4), 1–12. 10.1007/s40475-024-00326-y

[gh270085-bib-0029] Sattari, A. , Jafarzadegan, K. , & Moradkhani, H. (2024). Enhancing streamflow predictions with machine learning and copula‐embedded Bayesian model averaging. Journal of Hydrology, 643, 131986. 10.1016/j.jhydrol.2024.131986

[gh270085-bib-0030] Scallan, E. , Hoekstra, R. M. , Angulo, F. J. , Tauxe, R. V. , Widdowson, M.‐A. , Roy, S. L. , et al. (2011). Foodborne illness acquired in the united states—Major pathogens. Emerging Infectious Diseases, 17(1), 7–15. 10.3201/eid1701.p11101 21192848 PMC3375761

[gh270085-bib-0031] Schramm, P. J. , Ahmed, M. , Siegel, H. , Donatuto, J. , Campbell, L. , Raab, K. , & Svendsen, E. (2020). Climate change and health: Local solutions to local challenges. Current Environmental Health Reports, 7(4), 363–370. 10.1007/s40572-020-00294-1 33113083 PMC7591693

[gh270085-bib-0032] Schwarz, G. (1978). Estimating the dimension of a model. Annals of Statistics, 6(2), 461–464. 10.1214/aos/1176344136

[gh270085-bib-0033] Semenza, J. C. , & Menne, B. (2012). Climate change impact assessment of food‐ and waterborne diseases. Critical Reviews in Environmental Science and Technology, 42(7), 671–701. 10.1080/10643389.2010.534706 PMC3996521 24808720

[gh270085-bib-0034] Simpson, V. , Thomas, J. , Brown, J. , Musto, J. , Graham, R. M. , Moore, M. , & Sintchenko, V. (2019). Divergent geography of *Salmonella* wangata and *Salmonella* typhimurium in new south wales, Australia. One Health, 7, 100092. 10.1016/j.onehlt.2019.100092 31016222 PMC6475636

[gh270085-bib-0035] Spiegelhalter, D. J. , Best, N. G. , Carlin, B. P. , & van der Linde, A. (2002). Bayesian measures of model complexity and fit. Journal of the Royal Statistical Society: Series B, 64(4), 583–639. 10.1111/1467-9868.00353

[gh270085-bib-0036] Tack, B. , Vita, D. , Phoba, M.‐F. , Mbuyi‐Kalonji, L. , Hardy, L. , Barbé, B. , et al. (2021). Direct association between rainfall and non‐typhoidal salmonella bloodstream infections in hospital‐admitted children in the democratic republic of Congo. Scientific Reports, 11(1), 21617. 10.1038/s41598-021-01030-x 34732799 PMC8566593

[gh270085-bib-0037] Tan, J. , Kalkstein, L. , Huang, J. , Lin, S. , Yin, H. , & Shao, D. (2004). An operational heat/health warning system in shanghai. International Journal of Biometeorology, 48(3), 157–162. 10.1007/s00484-003-0193-z 14586669

[gh270085-bib-0038] Thindwa, D. , Chipeta, M. G. , Henrion, M. Y. , & Gordon, M. A. (2019). Distinct climate influences on the risk of typhoid compared to invasive non‐typhoid salmonella disease in Blantyre, Malawi. Scientific Reports, 9(1), 20310. 10.1038/s41598-019-56688-1 31889080 PMC6937328

[gh270085-bib-0040] Wang, X. , Deng, C. , Yin, X. , Wei, J. , & Zou, J. C. (2025). Assessing climate change impacts on streamflow in upper han river basin using deep learning models ensembled with Bayesian model averaging. (In press). Water Science and Engineering, 18(4), 412–421. 10.1016/j.wse.2025.08.004

[gh270085-bib-0041] Williams, L. A. , Smith, J. D. , Brown, L. A. , & Wilson, H. (2020). Effects of ambient temperature on salmonella infections in temperate regions: A multi‐region time‐series analysis. Environmental Research, 182, 109034.

[gh270085-bib-0042] World Health Organization . (2018a). Climate change and health: Key facts. Retrieved from https://www.who.int/news‐room/fact‐sheets/detail/climate‐change‐and‐health(Factsheet

[gh270085-bib-0043] World Health Organization . (2018b). Salmonella (non‐typhoidal). Retrieved from https://www.who.int/news‐room/fact‐sheets/detail/salmonella‐(non‐typhoidal) (Accessed: 2025‐04‐09)

[gh270085-bib-0044] Zhang, Y. , Peng, B. , & Hiller, J. (2010). Climate variations and Salmonella infection in Australian subtropical and tropical regions. Science of the Total Environment, 408(3), 524–530. 10.1016/j.scitotenv.2009.10.068 19922981

