# Supplementary material for: Too Hot, Too Wet: Bayesian Spatial Modeling of Climate‐Driven Salmonella Risk in New South Wales, Australia, 1991–2022
Source: Geohealth. 2026 Jan 28;10(1):e2025GH001617. doi: 10.1029/2025GH001617 (PMC12848793; doi:10.1029/2025GH001617)
Supplement: Supplementary file 1 — Supporting Information S1 [file GH2-10-e2025GH001617-s001.pdf]

Supplementary Material to Too Hot, Too Wet: Bayesian Spatial Modelling of Climate-Driven Salmonella Risk in New South Wales, Australia, 1991–2022

Oyelola A. Adegboye[1,2]*†, Tehan Amarasena [2]†, Mohammad Afzal Khan[3]†, Hassan Ajulo[2], Anton Pak[4], David Taniar[3]Theophilus I. Emeto[2]

[1]Menzies School of Health Research, Darwin, Charles Darwin University, NT, Australia
[2]Public Health and Tropical Medicine, College of Medicine and Dentistry, James Cook University, Townsville, QLD 4811, Australia
[3]Faculty of Information Technology, Monash University, Melbourne, Australia
[4]Centre for the Business and Economics of Health, The University of Queensland, Brisbane, Queensland, Australia

S.1 Model assessment

Table S.1: Model fit and predictive performance metrics for case-crossover vs BSM.

Metric	CCM	BSM
RMSE (Root Mean Squared Error)	5.55	5.10
NRMSE (normalized)	0.416	0.382
R^2 (pseudo)	0.827	0.854
MAE (Mean Absolute Error)	3.46	3.01
Symmetric Mean Absolute Percentage Error (SMAPE) (% error)	33.28	29.43
AIC (Akaike Information Criterion)	32395	31335
BIC (Bayesian Information Criterion)	32422	31361
DIC	1.217×10^6	9.980×10^5
QAIC (quasi-likelihood AIC)	**1058.7**	1212.3
$\text{QAIC}_{\text{MCMC}}$ (Bayesian)	2.401×10^6	1.963×10^6
Overdispersion ϕ	30.825	26.012

Corresponding author: Oyelola Adegboye, oyelola.adegboye@menzies.edu.au

14 ## S.2 Exploratory Data Analysis

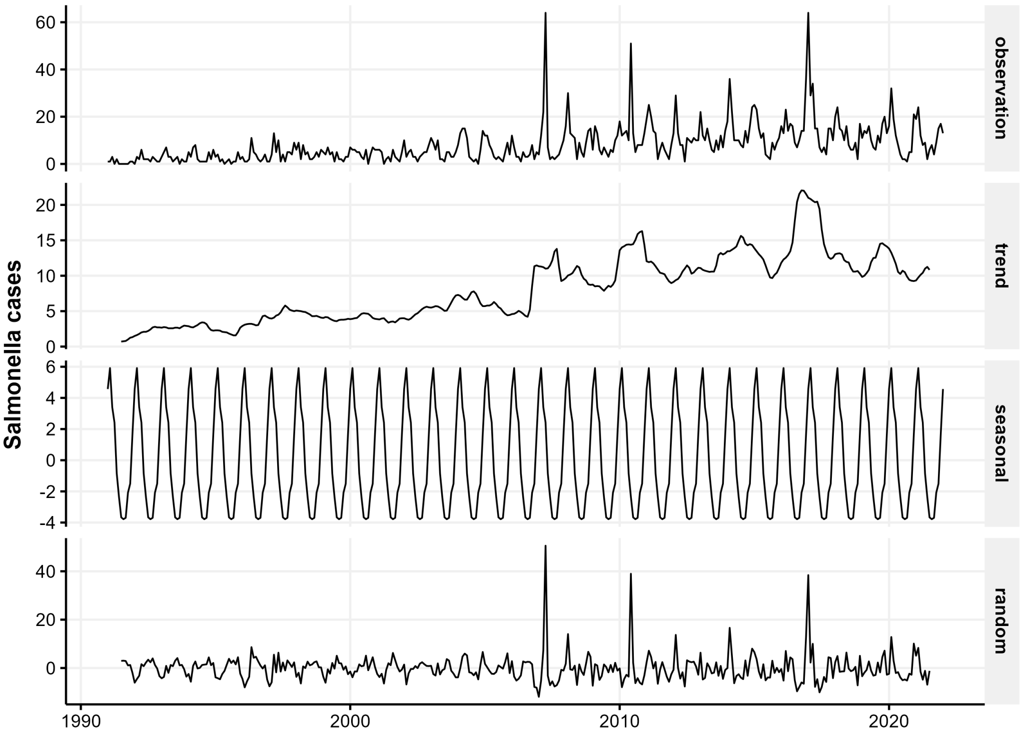

Figure S.1: Decomposition of *Salmonella* time series data

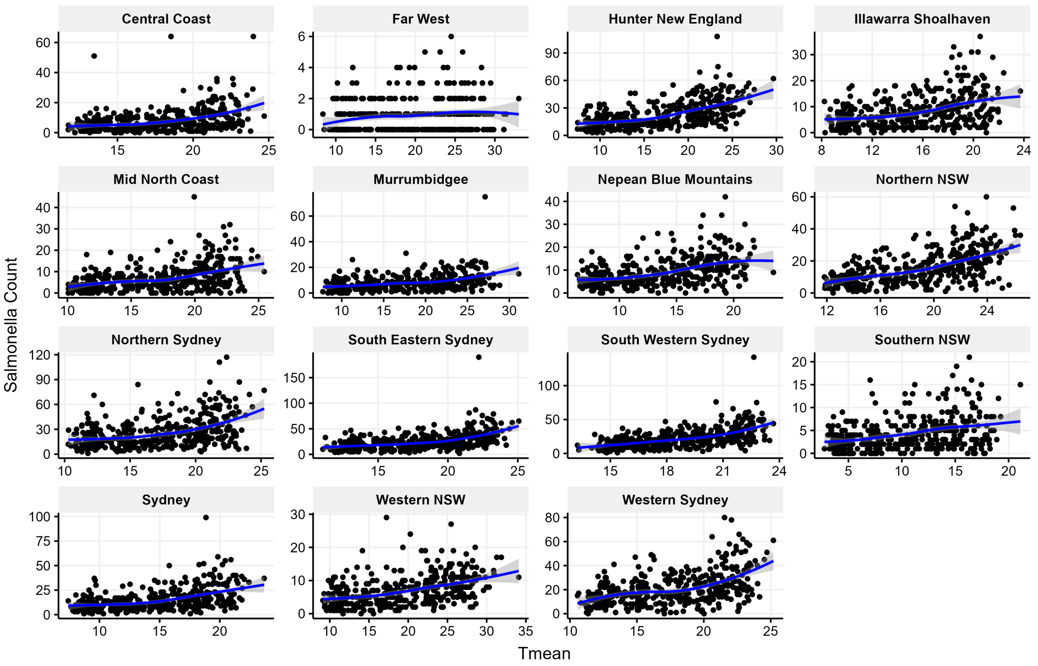

Figure S.2: *Salmonella* cases vs monthly mean temperature across the LHDs

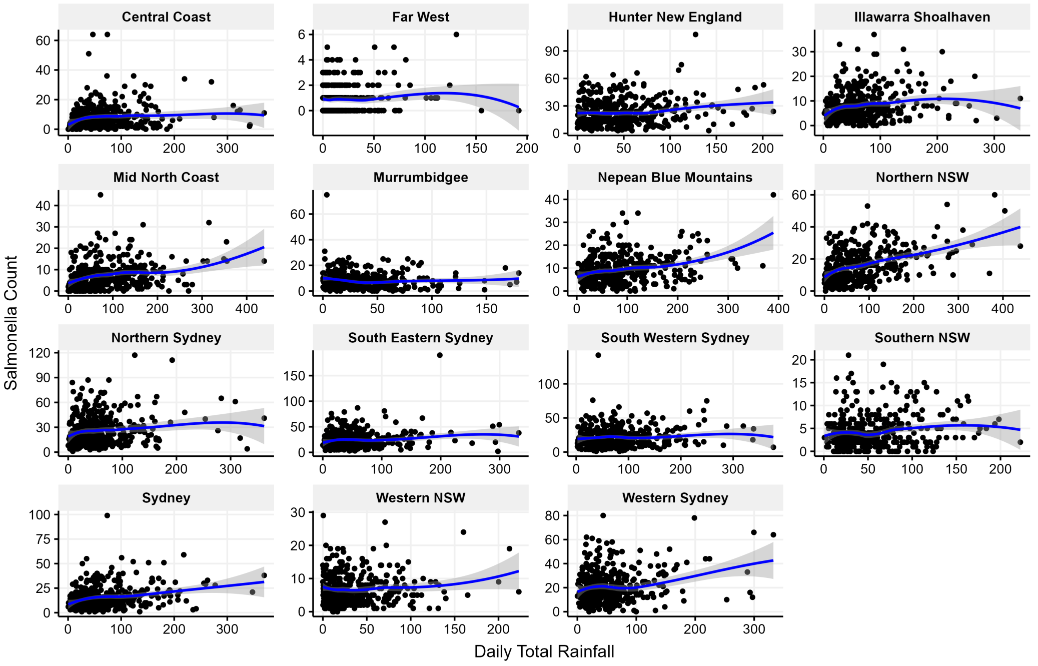

Figure S.3: *Salmonella* cases vs rainfall across the LHDs

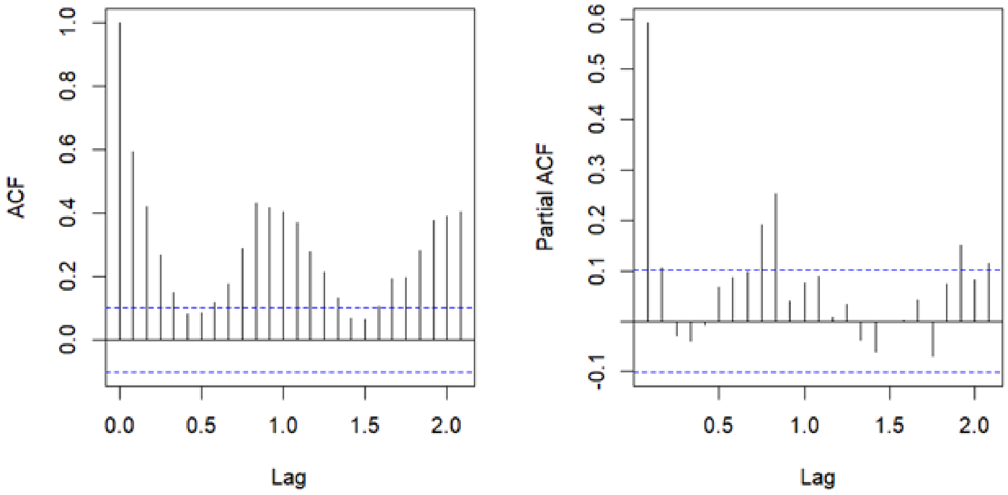

Figure S.4: Autocorrelation function (ACF) and partial ACF of Salmonella incidence rate

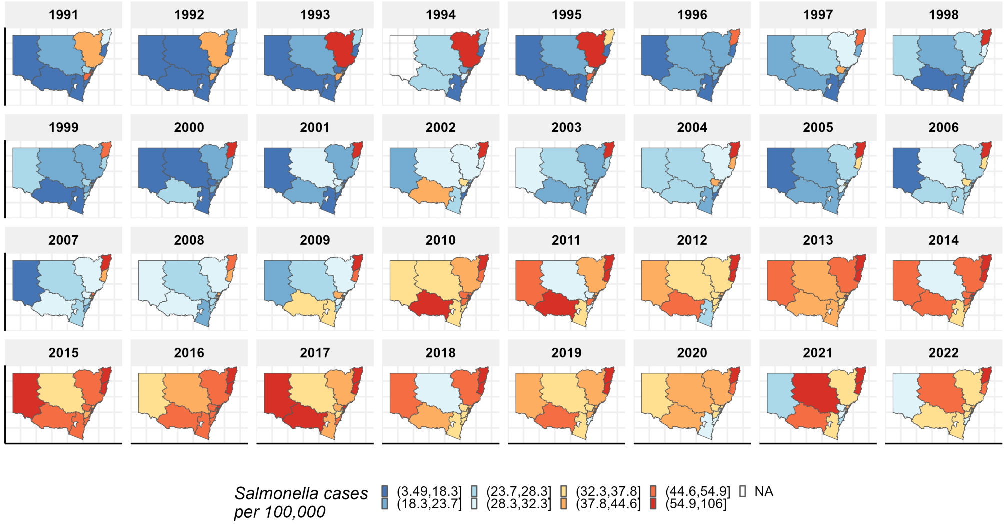

Figure S.5: Spatiotemporal patterns of *Salmonella* cases per 100,000 population across the LHDs in New South Wales, Australia, from 1990 to 2022

S.3 Dose-response curves

Supplementary Figures S6–S18: Spatiotemporal and climatic associations of *Salmonella* cases across Local Health Districts in New South Wales, Australia, using case-crossover and time-series models. Each panel shows district-level relative risk estimates and lag–response patterns under varying climatic conditions.

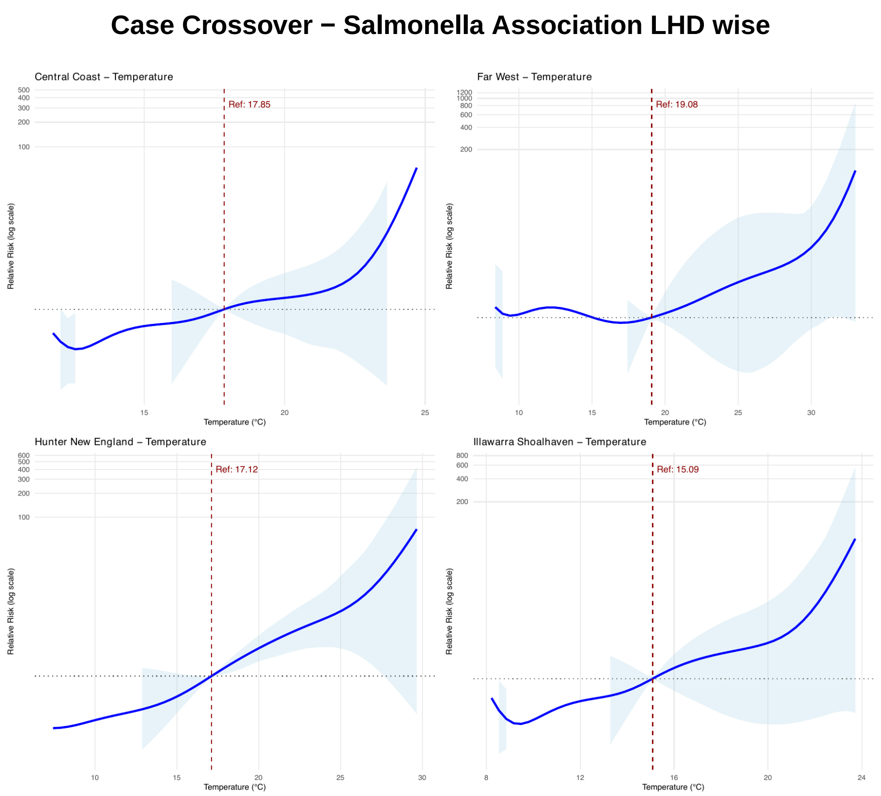
Case Crossover − Salmonella Association LHD wise
Central Coast – Temperature
Ref: 17.85
Far West – Temperature
Ref: 19.08
Hunter New England – Temperature
Ref: 17.12
Illawarra Shoalhaven – Temperature
Ref: 15.09
Relative Risk (log scale)
Temperature (°C)

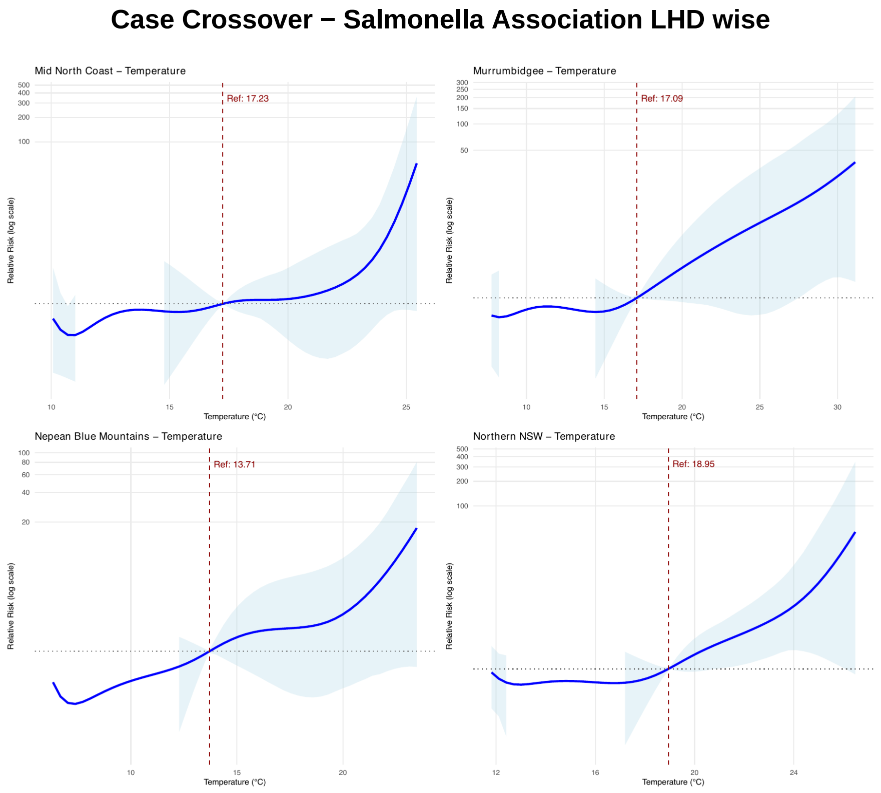
Case Crossover − Salmonella Association LHD wise
Mid North Coast – Temperature
Ref: 17.23
Murrumbidgee – Temperature
Ref: 17.09
Nepean Blue Mountains – Temperature
Ref: 13.71
Northern NSW – Temperature
Ref: 18.95
Relative Risk (log scale)
Temperature (°C)

Figure S.6

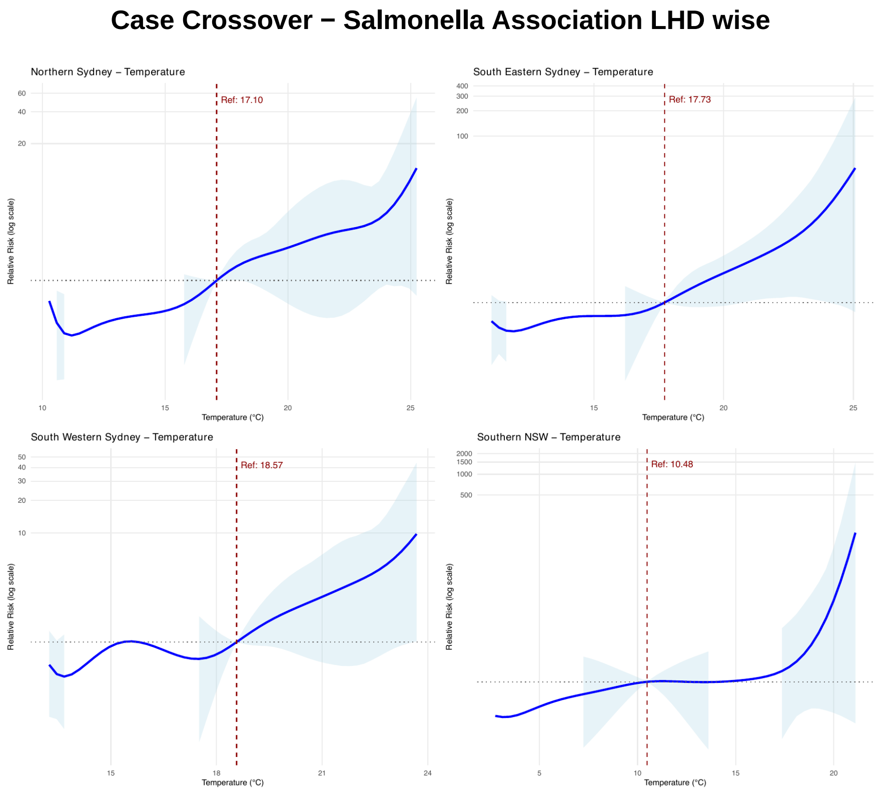
Case Crossover − Salmonella Association LHD wise
Northern Sydney – Temperature
Ref: 17.10
South Eastern Sydney – Temperature
Ref: 17.73
South Western Sydney – Temperature
Ref: 18.57
Southern NSW – Temperature
Ref: 10.48
Relative Risk (log scale)
Temperature (°C)

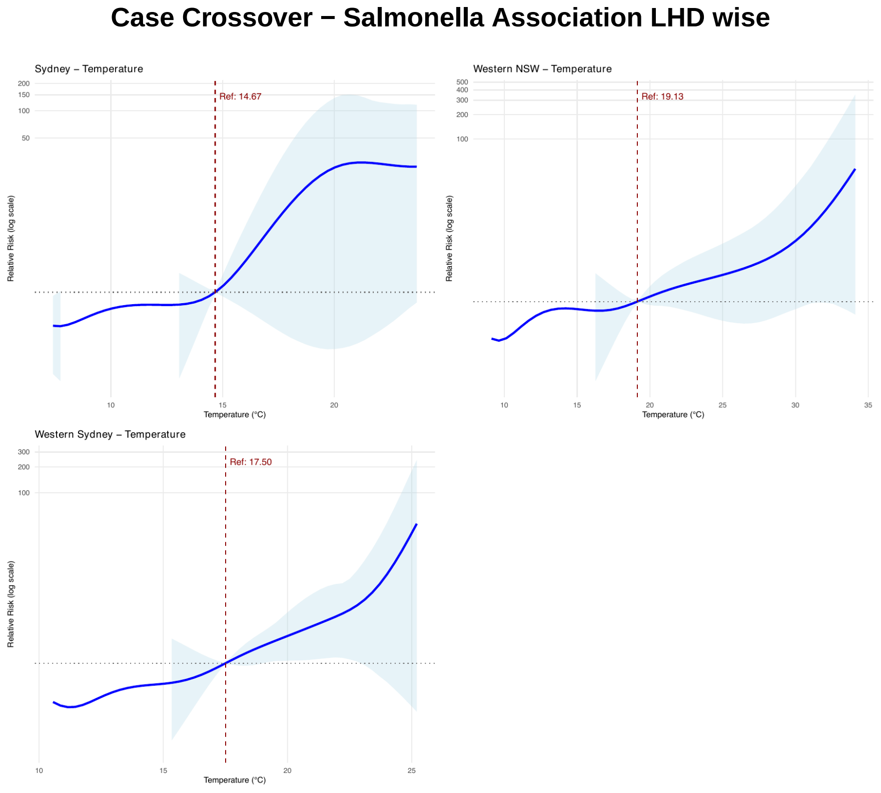
Case Crossover − Salmonella Association LHD wise
Sydney – Temperature
Ref: 14.67
Western NSW – Temperature
Ref: 19.13
Western Sydney – Temperature
Ref: 17.50
Relative Risk (log scale)
Temperature (°C)

Figure S.7

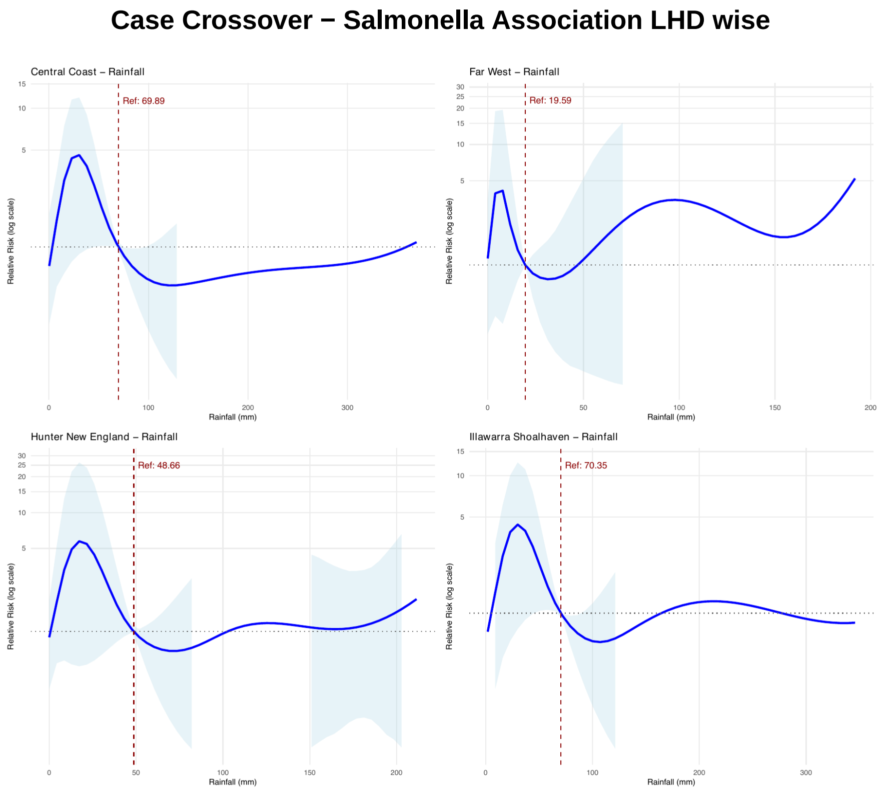
Case Crossover − Salmonella Association LHD wise
Central Coast – Rainfall
Ref: 69.89
Far West – Rainfall
Ref: 19.59
Hunter New England – Rainfall
Ref: 48.66
Illawarra Shoalhaven – Rainfall
Ref: 70.35
Relative Risk (log scale)
Rainfall (mm)

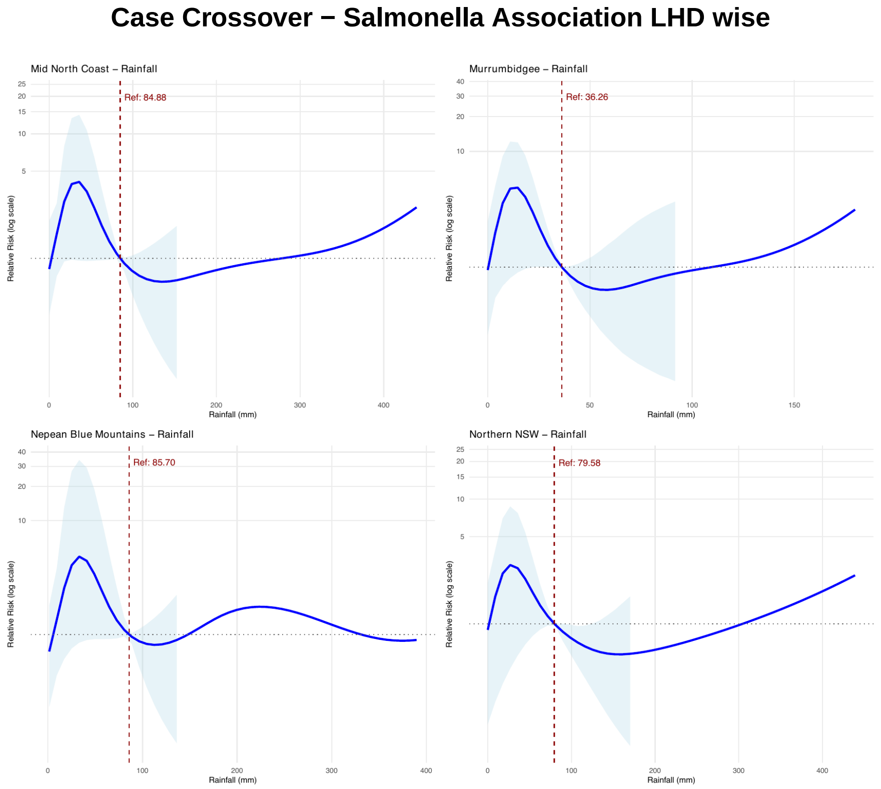
Case Crossover − Salmonella Association LHD wise
Mid North Coast – Rainfall
Ref: 84.88
Murrumbidgee – Rainfall
Ref: 36.26
Nepean Blue Mountains – Rainfall
Ref: 85.70
Northern NSW – Rainfall
Ref: 79.58
Relative Risk (log scale)
Rainfall (mm)

Figure S.8

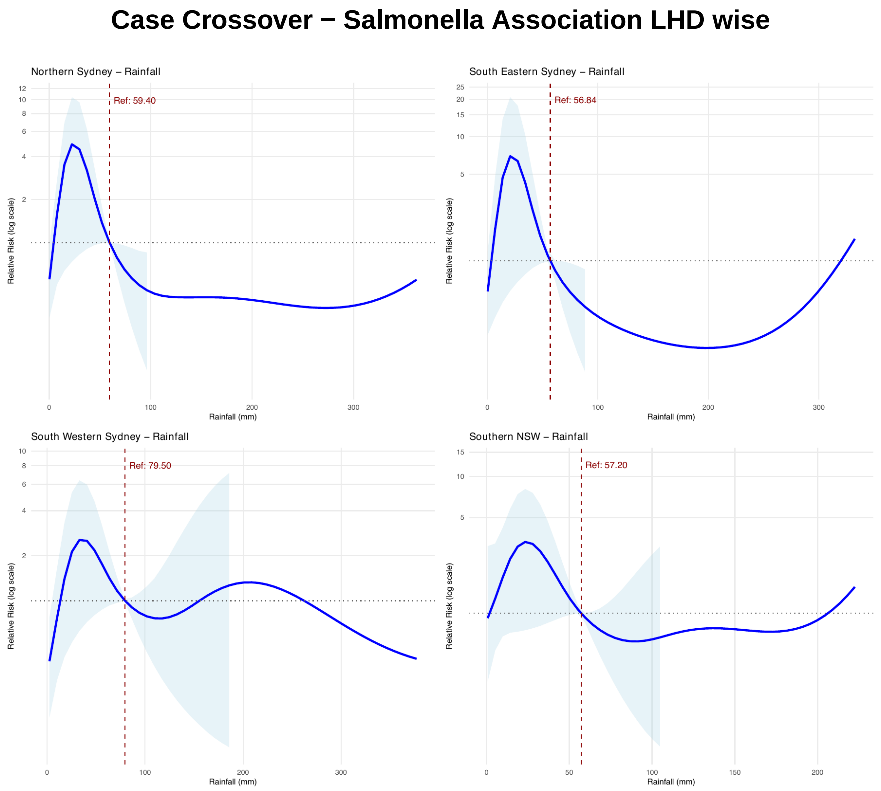
Case Crossover − Salmonella Association LHD wise
Northern Sydney – Rainfall
Ref: 59.40
South Eastern Sydney – Rainfall
Ref: 56.84
South Western Sydney – Rainfall
Ref: 79.50
Southern NSW – Rainfall
Ref: 57.20
Relative Risk (log scale)
Rainfall (mm)

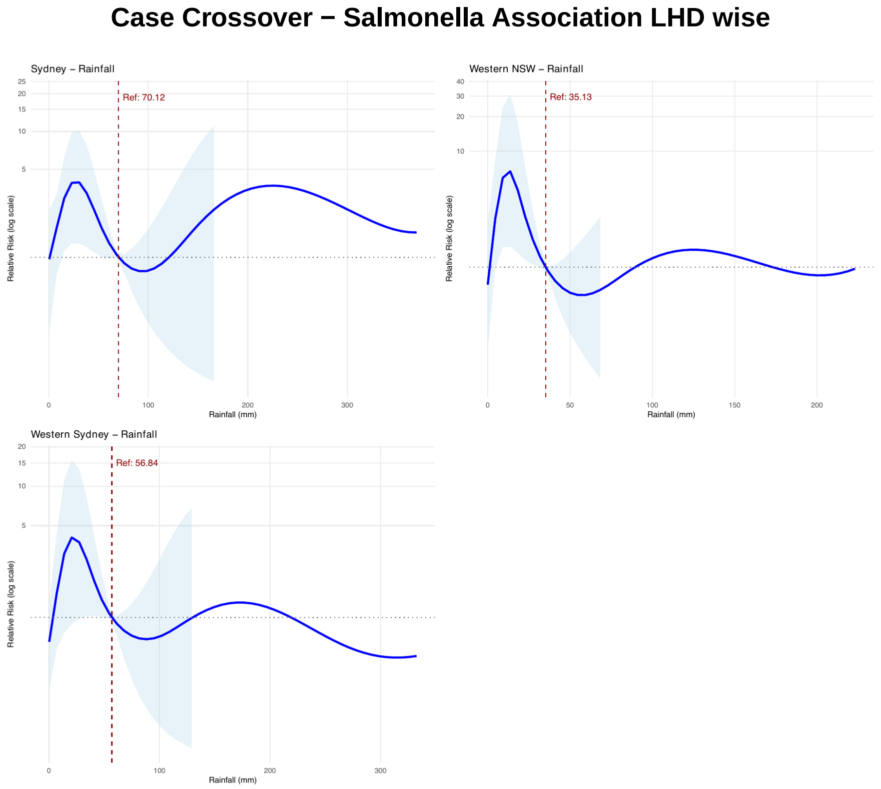
Case Crossover − Salmonella Association LHD wise
Sydney – Rainfall
Ref: 70.12
Western NSW – Rainfall
Ref: 35.13
Western Sydney – Rainfall
Ref: 56.84
Relative Risk (log scale)
Rainfall (mm)

Figure S.9

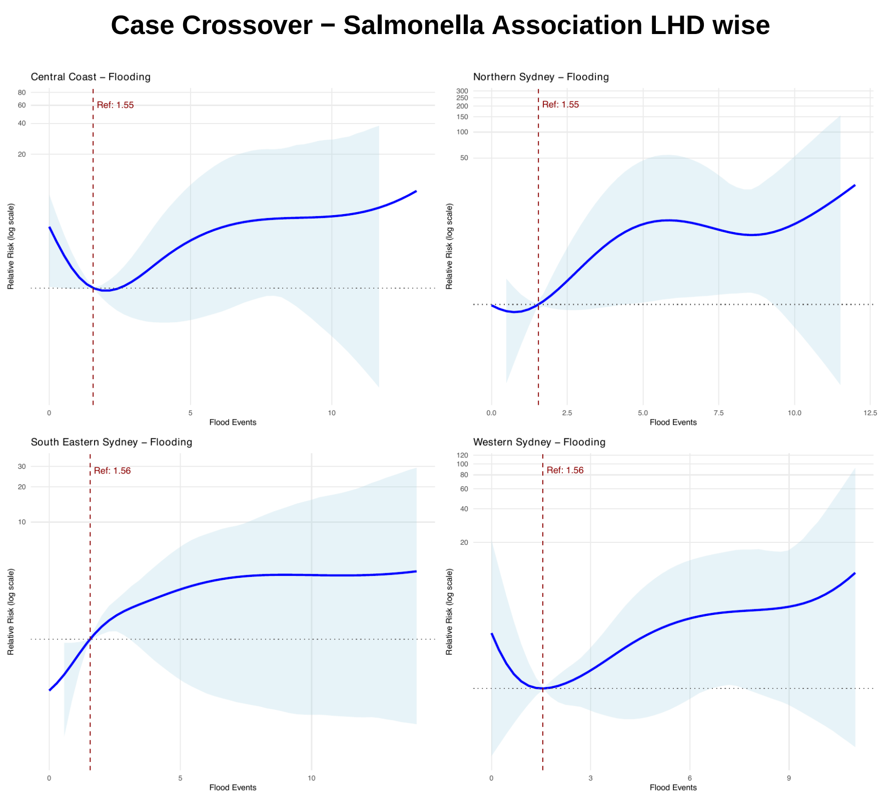
Case Crossover − Salmonella Association LHD wise
Central Coast – Flooding
Ref: 1.55
Northern Sydney – Flooding
Ref: 1.55
South Eastern Sydney – Flooding
Ref: 1.56
Western Sydney – Flooding
Ref: 1.56
Relative Risk (log scale)
Flood Events

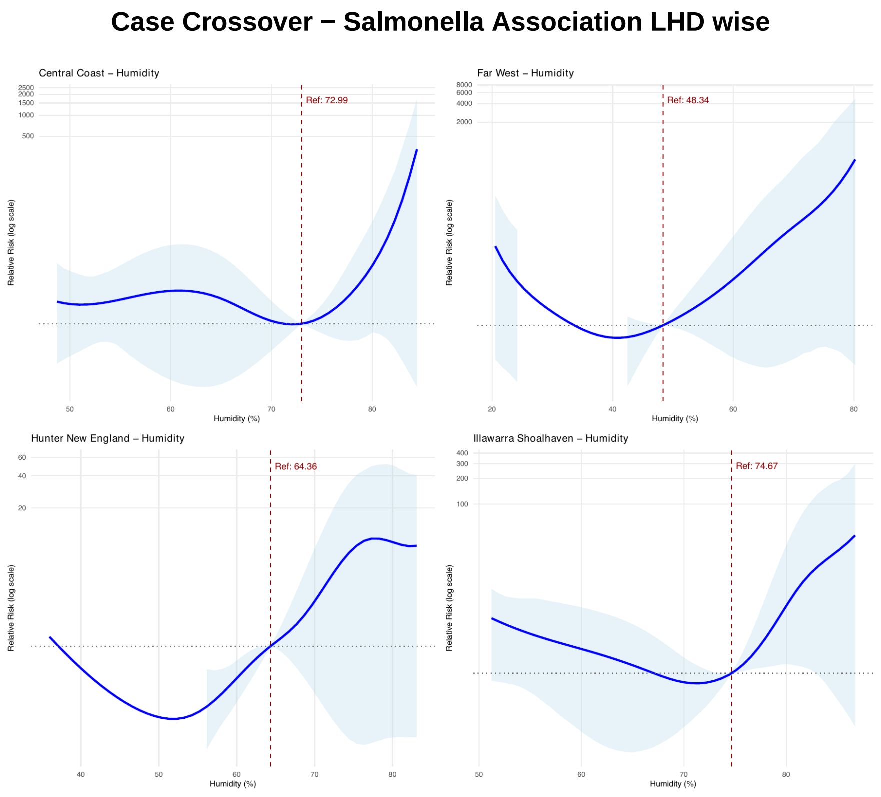
Case Crossover − Salmonella Association LHD wise
Central Coast – Humidity
Ref: 72.99
Far West – Humidity
Ref: 48.34
Hunter New England – Humidity
Ref: 64.36
Illawarra Shoalhaven – Humidity
Ref: 74.67
Relative Risk (log scale)
Humidity (%)

Figure S.10

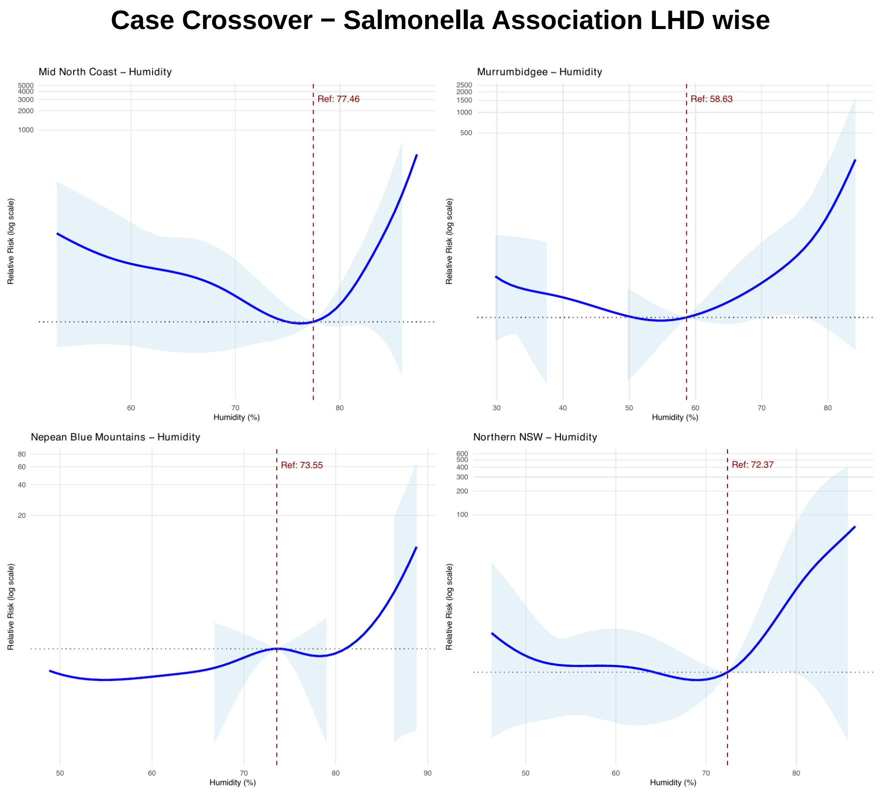
Case Crossover − Salmonella Association LHD wise
Mid North Coast – Humidity
Ref: 77.46
Murrumbidgee – Humidity
Ref: 58.63
Nepean Blue Mountains – Humidity
Ref: 73.55
Northern NSW – Humidity
Ref: 72.37
Relative Risk (log scale)
Humidity (%)

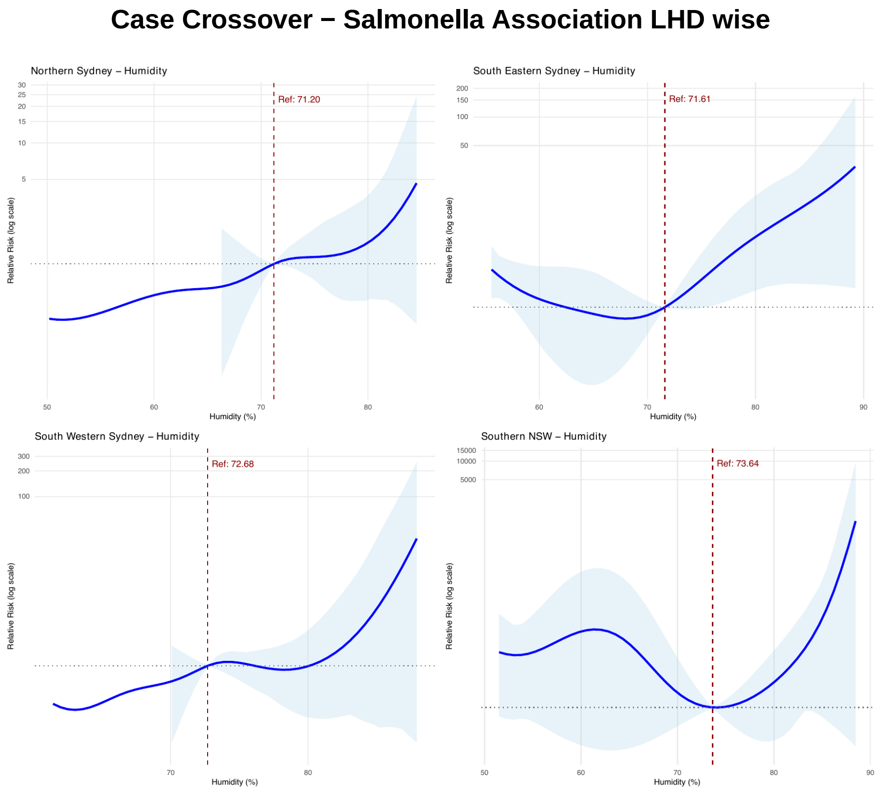
Case Crossover − Salmonella Association LHD wise
Northern Sydney – Humidity
Ref: 71.20
South Eastern Sydney – Humidity
Ref: 71.61
South Western Sydney – Humidity
Ref: 72.68
Southern NSW – Humidity
Ref: 73.64
Relative Risk (log scale)
Humidity (%)

Figure S.11

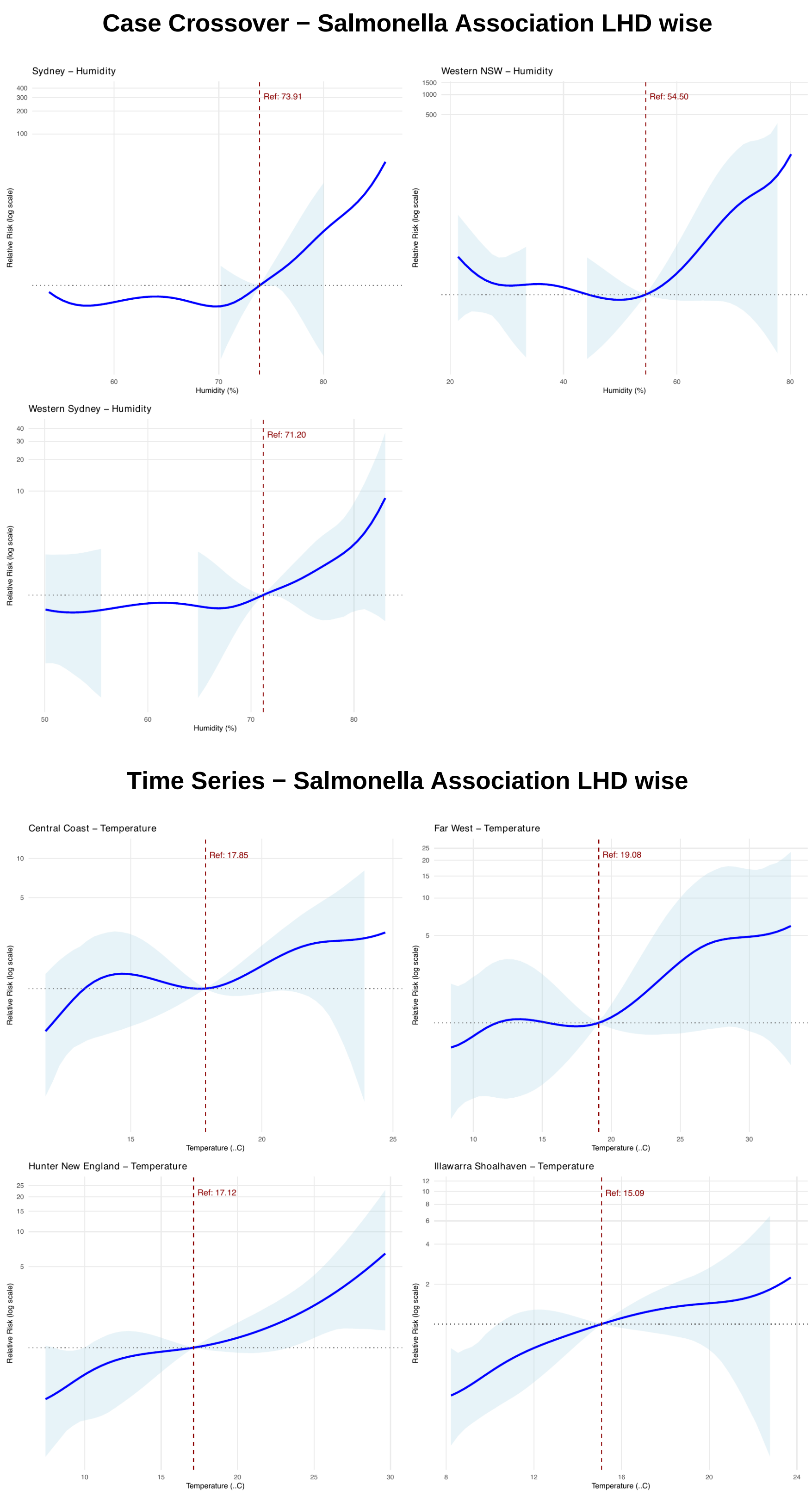
Case Crossover − Salmonella Association LHD wise
Sydney – Humidity
Ref: 73.91
Western NSW – Humidity
Ref: 54.50
Western Sydney – Humidity
Ref: 71.20
Relative Risk (log scale)
Humidity (%)
Time Series − Salmonella Association LHD wise
Central Coast – Temperature
Ref: 17.85
Far West – Temperature
Ref: 19.08
Hunter New England – Temperature
Ref: 17.12
Illawarra Shoalhaven – Temperature
Ref: 15.09
Relative Risk (log scale)
Temperature (..C)

Figure S.12

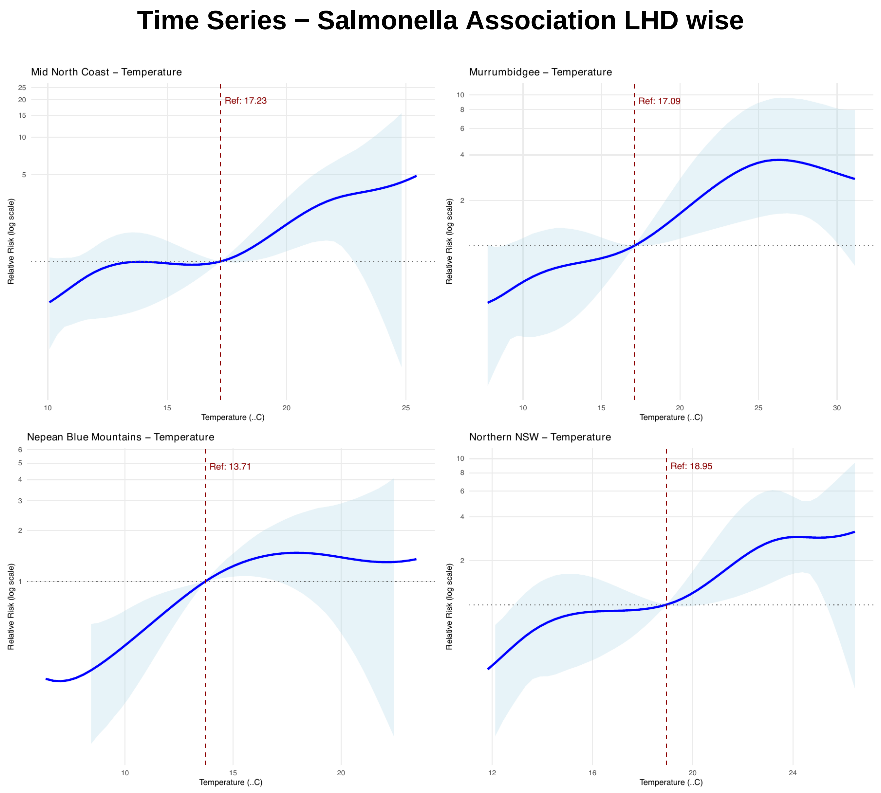
Time Series − Salmonella Association LHD wise
Mid North Coast – Temperature
Ref: 17.23
Murrumbidgee – Temperature
Ref: 17.09
Nepean Blue Mountains – Temperature
Ref: 13.71
Northern NSW – Temperature
Ref: 18.95
Relative Risk (log scale)
Temperature (..C)

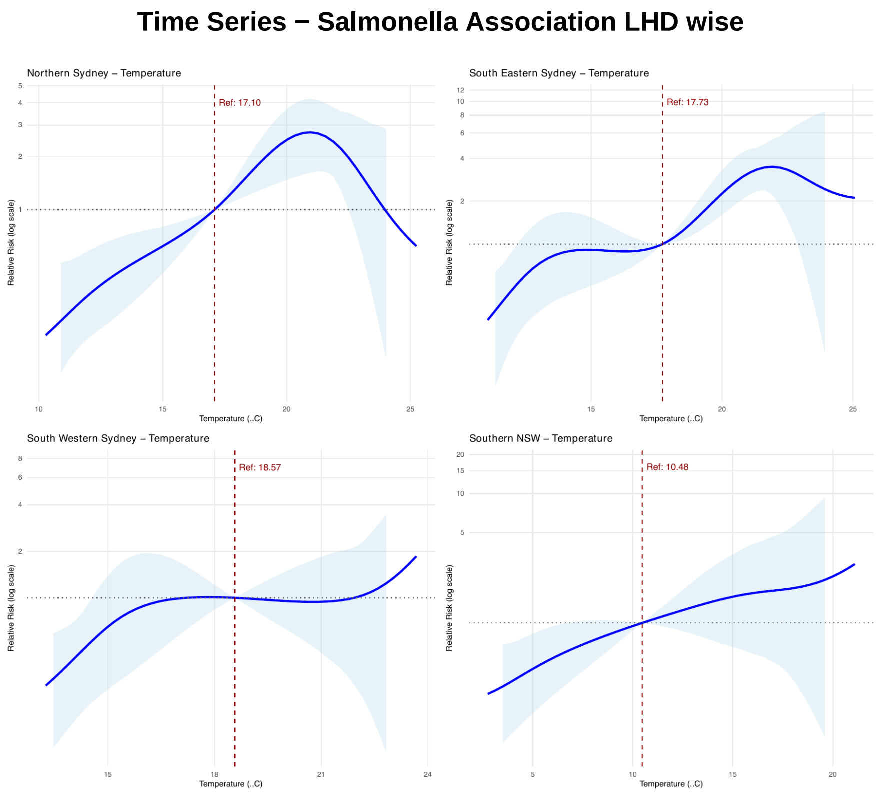
Time Series − Salmonella Association LHD wise
Northern Sydney – Temperature
Ref: 17.10
South Eastern Sydney – Temperature
Ref: 17.73
South Western Sydney – Temperature
Ref: 18.57
Southern NSW – Temperature
Ref: 10.48
Relative Risk (log scale)
Temperature (..C)

Figure S.13

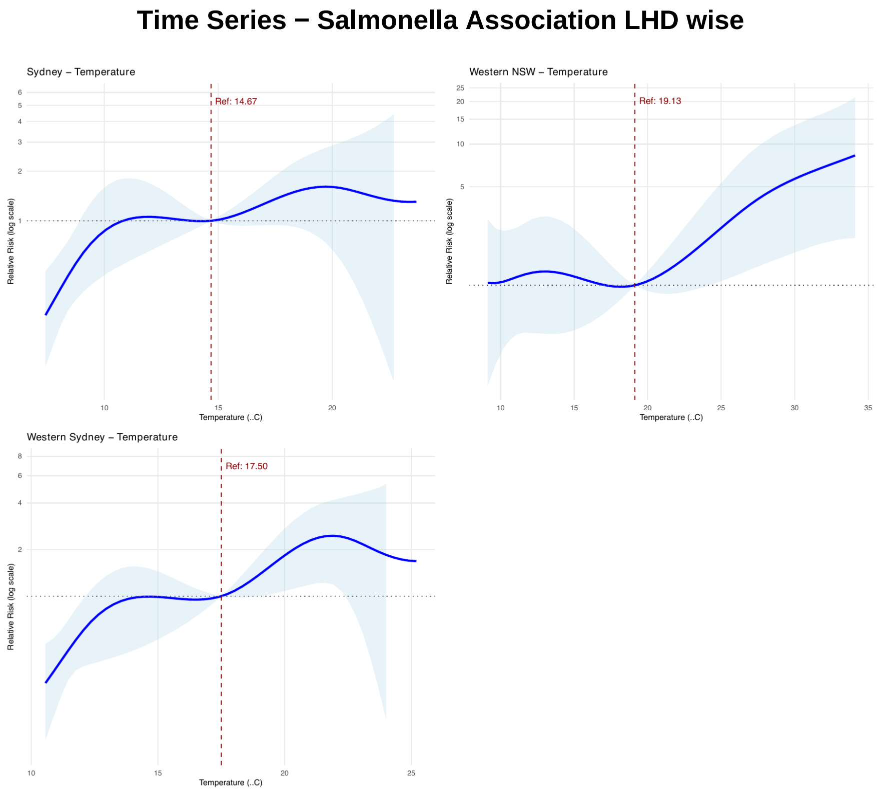
Time Series − Salmonella Association LHD wise
Sydney – Temperature
Ref: 14.67
Western NSW – Temperature
Ref: 19.13
Western Sydney – Temperature
Ref: 17.50
Relative Risk (log scale)
Temperature (..C)

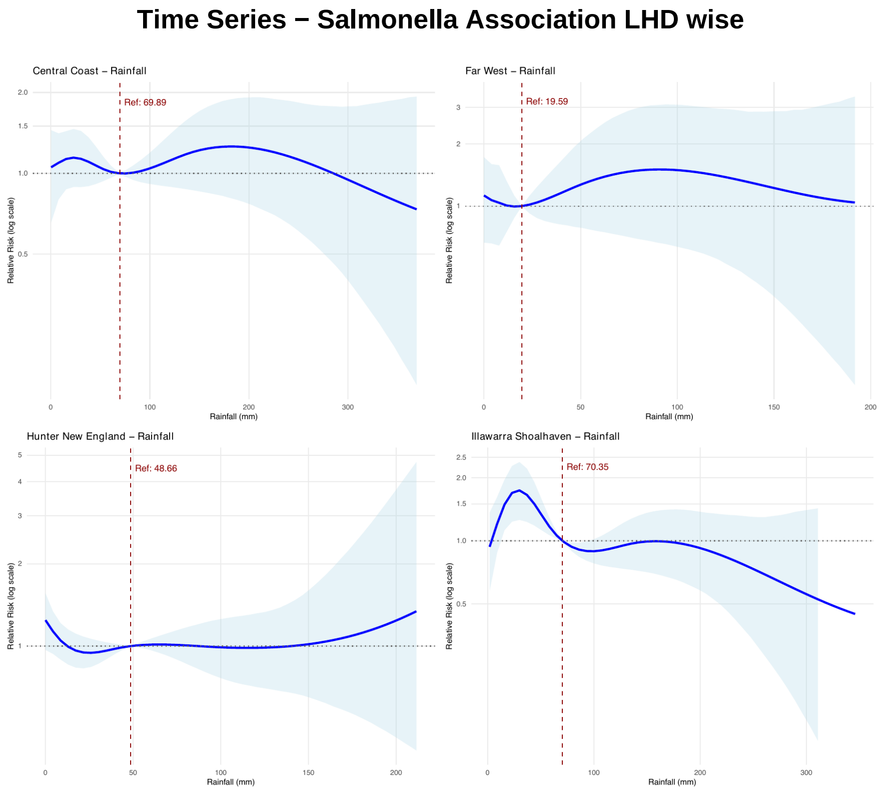
Time Series − Salmonella Association LHD wise
Central Coast – Rainfall
Ref: 69.89
Far West – Rainfall
Ref: 19.59
Hunter New England – Rainfall
Ref: 48.66
Illawarra Shoalhaven – Rainfall
Ref: 70.35
Relative Risk (log scale)
Rainfall (mm)

Figure S.14

Time Series − Salmonella Association LHD wise

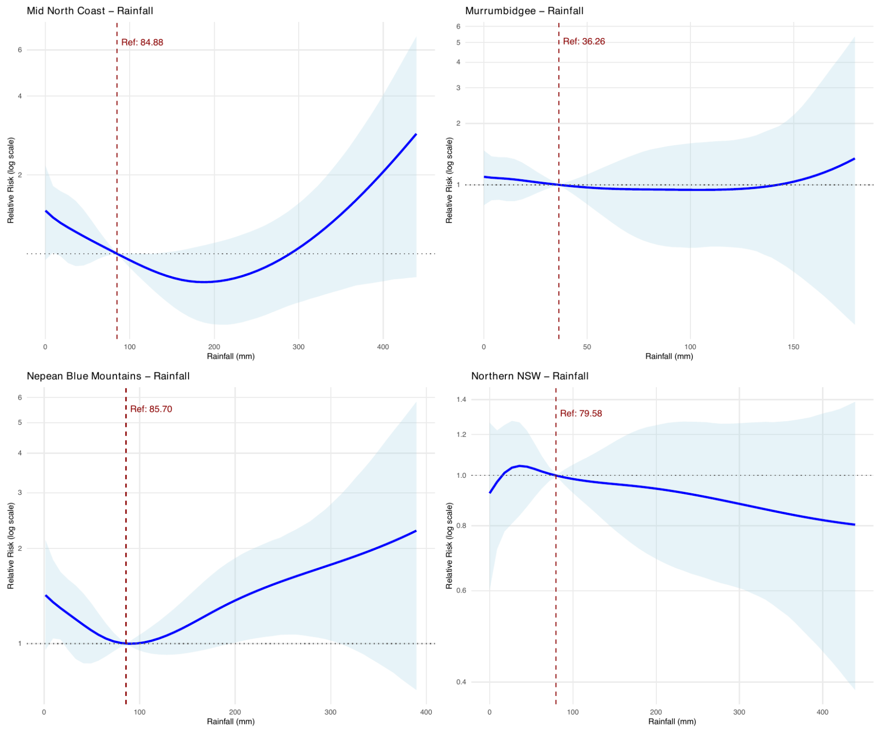

Time Series − Salmonella Association LHD wise

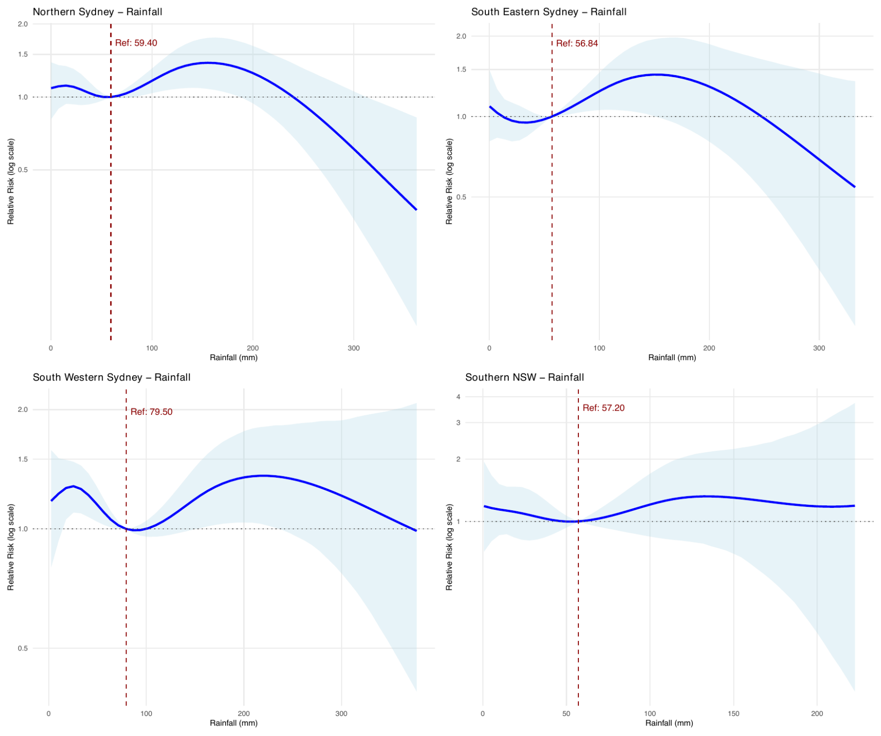

Figure S.15

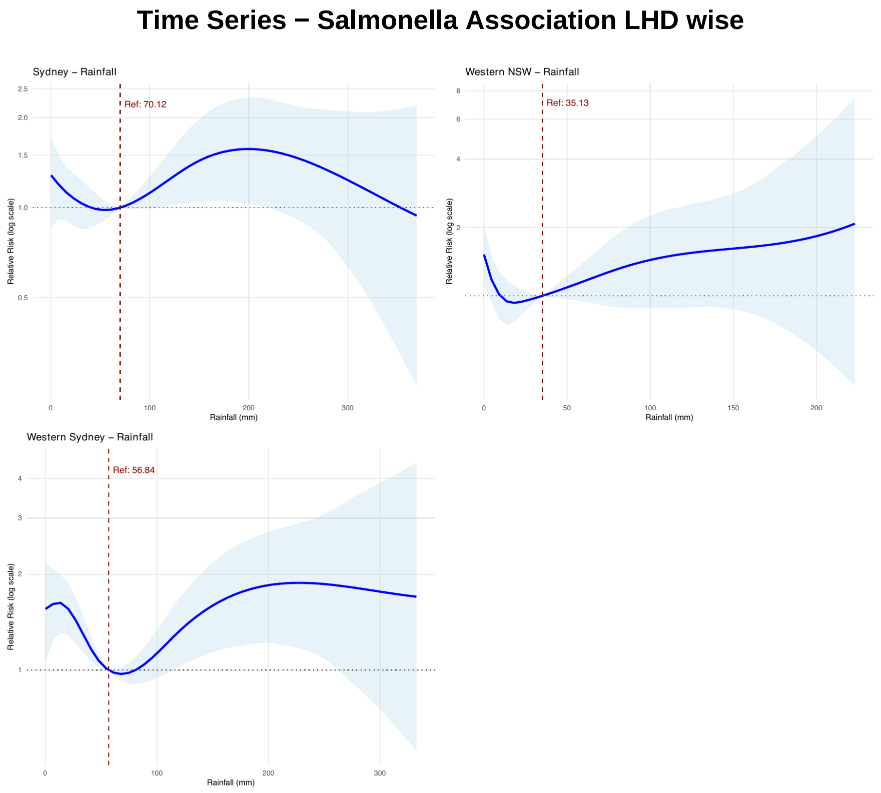
Time Series − Salmonella Association LHD wise
Sydney – Rainfall
Ref: 70.12
Western NSW – Rainfall
Ref: 35.13
Western Sydney – Rainfall
Ref: 56.84
Relative Risk (log scale)
Rainfall (mm)

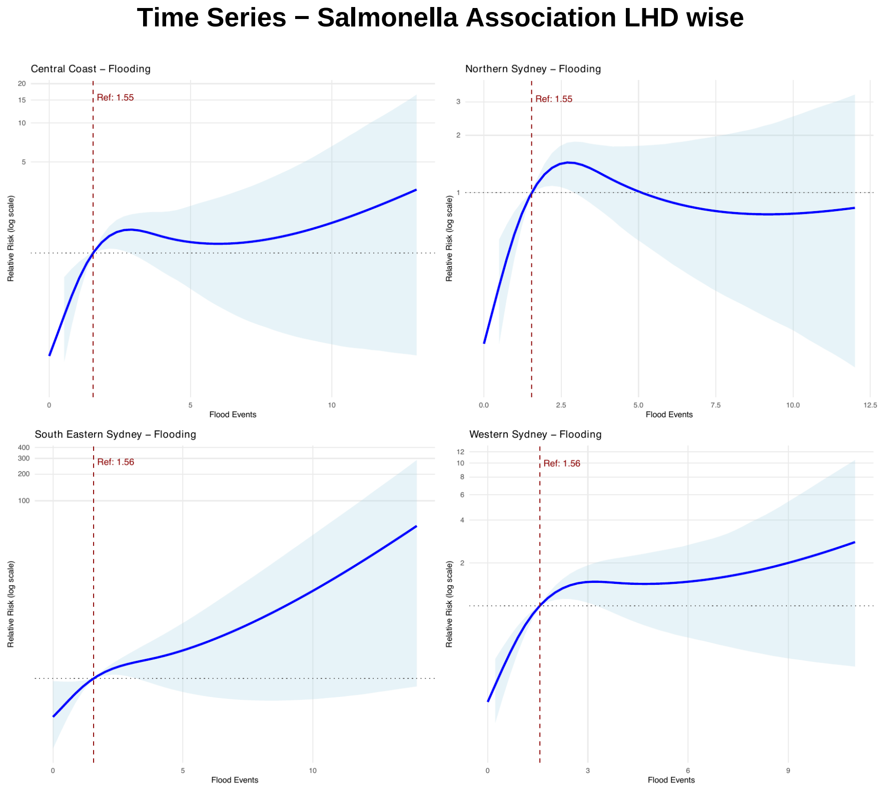
Time Series − Salmonella Association LHD wise
Central Coast – Flooding
Ref: 1.55
Northern Sydney – Flooding
Ref: 1.55
South Eastern Sydney – Flooding
Ref: 1.56
Western Sydney – Flooding
Ref: 1.56
Relative Risk (log scale)
Flood Events

Figure S.16

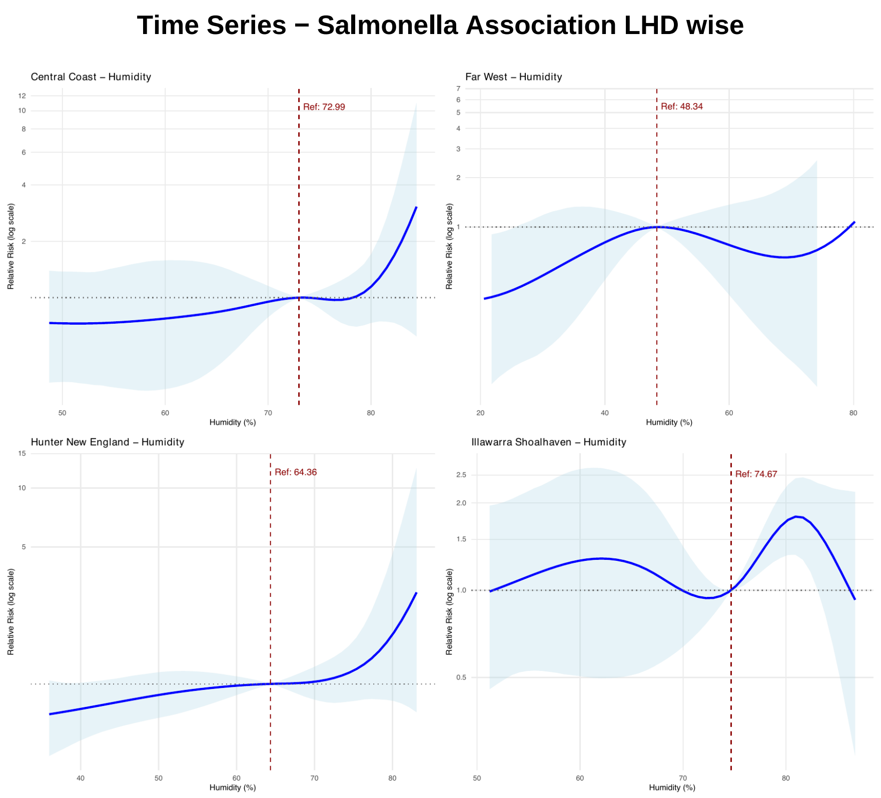
Time Series − Salmonella Association LHD wise
Central Coast – Humidity
Ref: 72.99
Far West – Humidity
Ref: 48.34
Hunter New England – Humidity
Ref: 64.36
Illawarra Shoalhaven – Humidity
Ref: 74.67
Relative Risk (log scale)
Humidity (%)

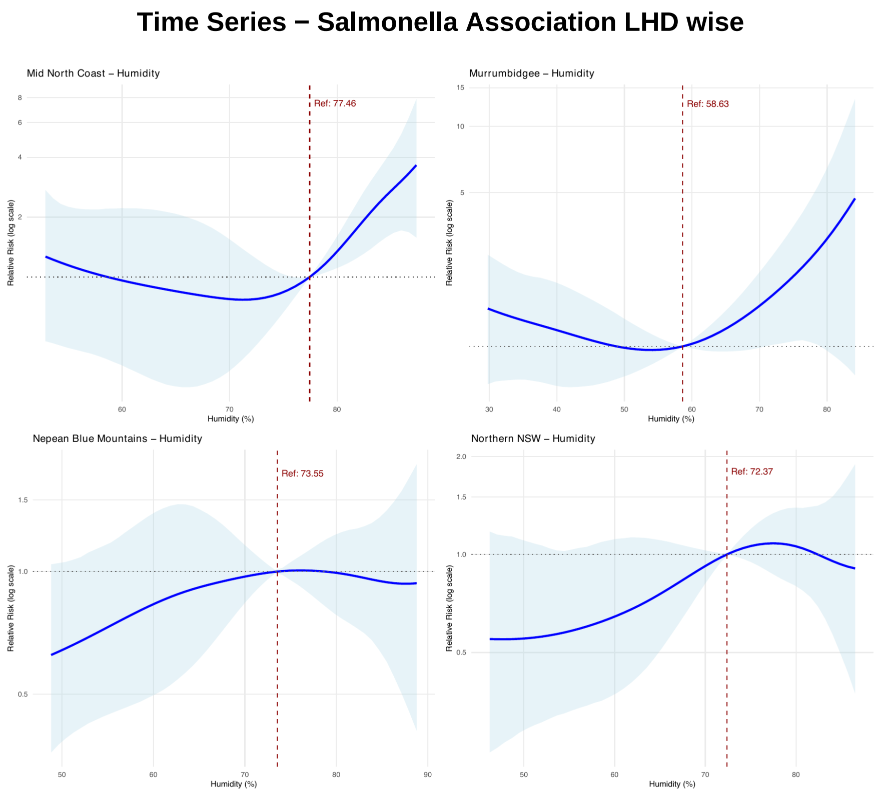
Time Series − Salmonella Association LHD wise
Mid North Coast – Humidity
Ref: 77.46
Murrumbidgee – Humidity
Ref: 58.63
Nepean Blue Mountains – Humidity
Ref: 73.55
Northern NSW – Humidity
Ref: 72.37
Relative Risk (log scale)
Humidity (%)

Figure S.17

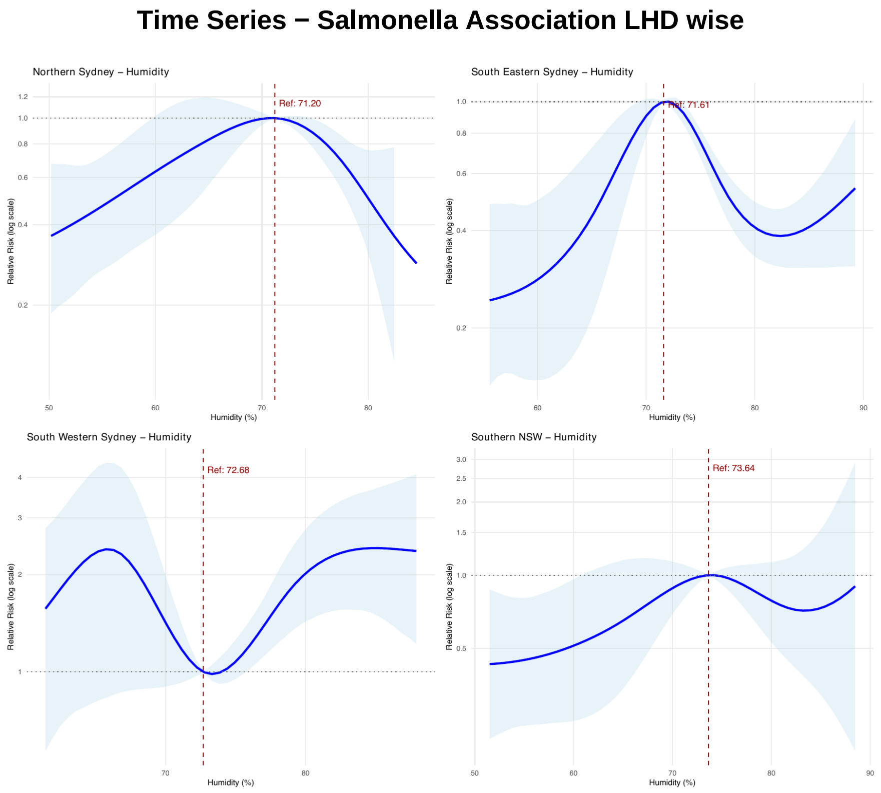
Time Series − Salmonella Association LHD wise
Northern Sydney – Humidity
Ref: 71.20
South Eastern Sydney – Humidity
Ref: 71.61
South Western Sydney – Humidity
Ref: 72.68
Southern NSW – Humidity
Ref: 73.64
Relative Risk (log scale)
Humidity (%)

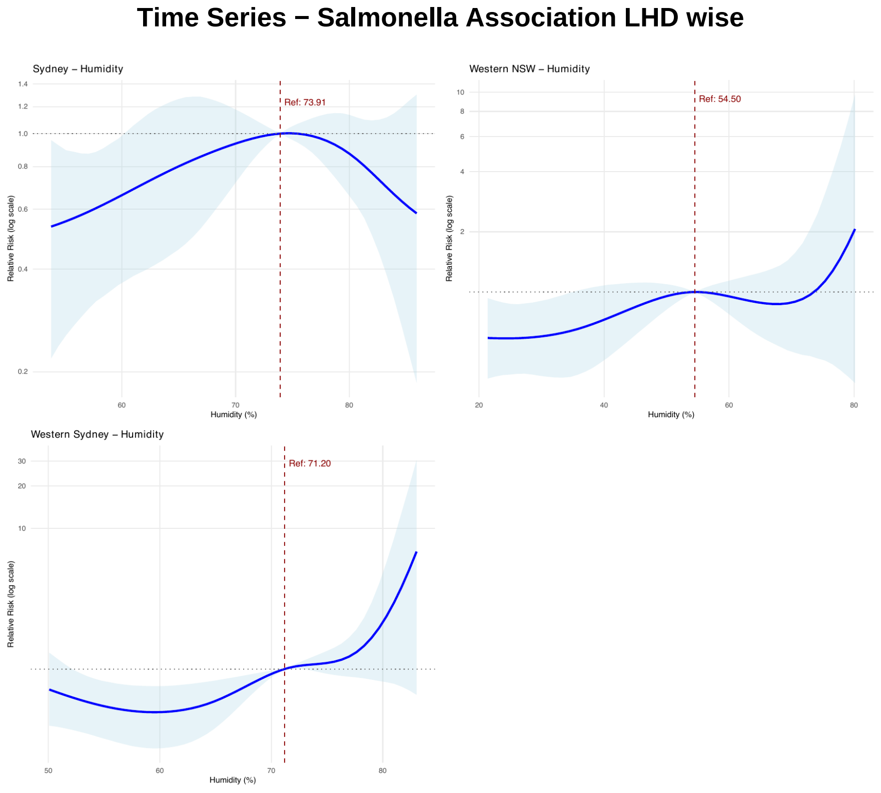
Time Series − Salmonella Association LHD wise
Sydney – Humidity
Ref: 73.91
Western NSW – Humidity
Ref: 54.50
Western Sydney – Humidity
Ref: 71.20
Relative Risk (log scale)
Humidity (%)

Figure S.18

S.4 Probability maps for case-crossover DLNM (CC)

Posterior probability that relative risk (RR) exceeds certain thresholds across NSW for Case-crossover DLNM.

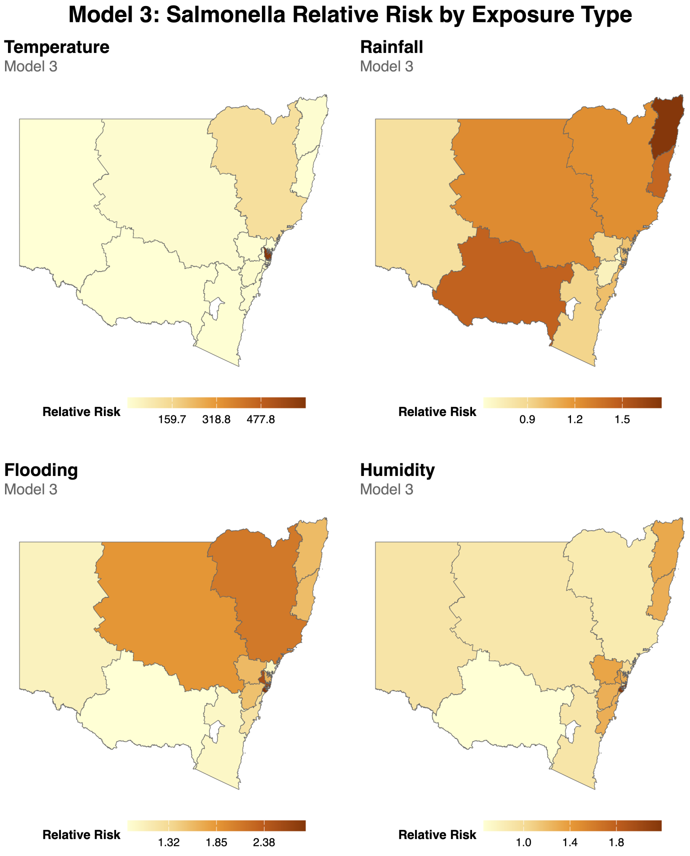

Figure S.19: Posterior probability map illustrating $P(\text{RR} > 1.0, 1.2, 1.5)$ under CC DLNM for different climate exposures. Darker colours indicate higher confidence that the relative risk surpasses the chosen threshold. Some LHDs exhibit distinct vulnerability to temperature, while others are more concerned with rainfall or flooding.

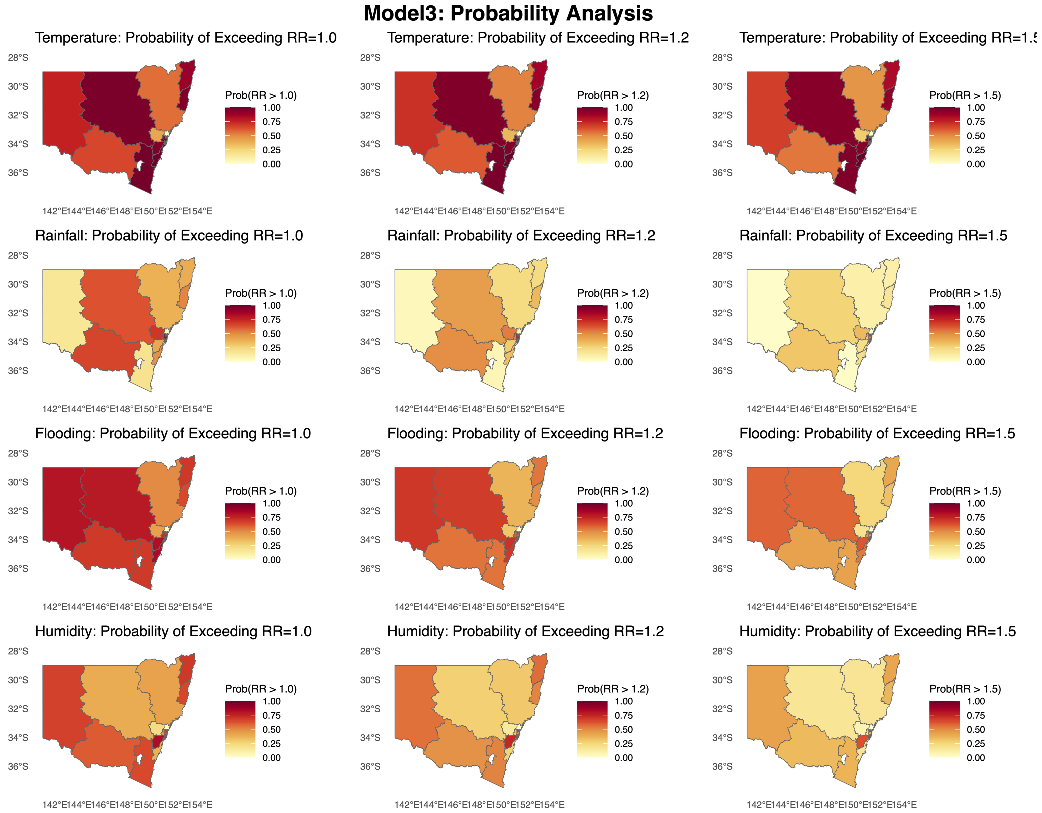

Figure S.20: An alternative or complementary probability map under CC-DLNM, possibly at different thresholds or for a different variable subset. These maps highlight localised patterns of exceedance risk, guiding targeted interventions in high-probability regions.
